# Supplementary material for: Prognostic impact of depressive symptoms on all-cause mortality in individuals with abdominal aortic aneurysm and in the general population: a population-based prospective HUNT study in Norway
Source: BMJ Open. 2022 Jan 15;12(1):e049055. doi: 10.1136/bmjopen-2021-049055 (PMC8765023; doi:10.1136/bmjopen-2021-049055)
Supplement: Supplementary data [file bmjopen-2021-049055supp001.pdf]

Supplemental material

The prognostic impact of depressive symptoms on all-cause mortality in individuals with abdominal aortic aneurysm and in the general population. A population-based prospective HUNT Study in Norway

Linn Å. Nyrønning[1,2] Rebecka Hultgren[3,4] Grethe Albrektsen[5,6], Erney Mattsson[1,2]

Malin Stenman[3,7]

[1]Department of Surgery, Vascular Surgery, St Olavs Hospital, Trondheim, Norway,

[2]Department of Circulation and Medical Imaging, NTNU, Trondheim, Norway,

[3]Department of Molecular Medicine and Surgery, Karolinska Institutet, Stockholm, Sweden,

[4]Department of Vascular Surgery, Karolinska University Hospital, Stockholm, Sweden,

[5]Department of Public Health and Nursing, NTNU, Trondheim, Norway,

[6]Department of clinical and molecular medicine, NTNU, Trondheim, Norway,

[7]Perioperative Medicine and Intensive Care Function, Karolinska University Hospital, Stockholm, Sweden

Corresponding author:

Malin Stenman

Perioperative Medicine and Intensive Care Function

Karolinska University Hospital / Karolinska Institutet

SE-171 76 Stockholm, Sweden

E-mail: Malin.Stenman@ki.se

Figure S1. “Log - log plot” to test the proportional hazards assumption. If the plotted lines are reasonably parallel, the proportional hazards assumption has not been violated.
