# Supplementary material for: Prognostic impact of depressive symptoms on all-cause mortality in individuals with abdominal aortic aneurysm and in the general population: a population-based prospective HUNT study in Norway
Source: BMJ Open. 2022 Jan 15;12(1):e049055. doi: 10.1136/bmjopen-2021-049055 (PMC8765023; doi:10.1136/bmjopen-2021-049055)
Supplement: Author's manuscript [file bmjopen-2021-049055.draft_revisions.pdf]

BMJ Open is committed to open peer review. As part of this commitment we make the peer review history of every article we publish publicly available.

When an article is published we post the peer reviewers' comments and the authors' responses online. We also post the versions of the paper that were used during peer review. These are the versions that the peer review comments apply to.

The versions of the paper that follow are the versions that were submitted during the peer review process. They are not the versions of record or the final published versions. They should not be cited or distributed as the published version of this manuscript.

BMJ Open is an open access journal and the full, final, typeset and author-corrected version of record of the manuscript is available on our site with no access controls, subscription charges or pay-per-view fees (http://bmjopen.bmj.com).

If you have any questions on BMJ Open's open peer review process please email info.bmjopen@bmj.com

BMJ Open

Mortality in individuals with Abdominal Aortic Aneurysm Do depressive symptoms worsen the prognosis? A HUNT Study

Journal:	*BMJ Open*
Manuscript ID	bmjopen-2021-049055
Article Type:	Original research
Date Submitted by the Author:	14-Jan-2021

Complete List of Authors:	Nyrønning, Linn Å.; Department of Surgery, Vascular Surgery; NTNU Department of Circulation and Medical Imaging<br>Hultgren, Rebecka; Karolinska Institute, Deparment of molecular medicine and surgery; Karolinska Universitetssjukhuset, Department of Vascular Surgery<br>Albrektsen, Grethe; Norges teknisk-naturvitenskapelige universitet, Department of public health and general practice<br>Mattsson, Erney; Department of Surgery, Vascular Surgery; NTNU Department of Circulation and Medical Imaging<br>Stenman, Malin ; Karolinska Institutet, Department of Molecular Medicine and Surgery; Karolinska University Hospital, Department of Molecular Medicine and Surgery

Keywords:	EPIDEMIOLOGY, Vascular medicine < INTERNAL MEDICINE, Vascular surgery < SURGERY, VASCULAR SURGERY

SCHOLARONE™
Manuscripts

I, the Submitting Author has the right to grant and does grant on behalf of all authors of the Work (as defined in the below author licence), an exclusive licence and/or a non-exclusive licence for contributions from authors who are: i) UK Crown employees; ii) where BMJ has agreed a CC-BY licence shall apply, and/or iii) in accordance with the terms applicable for US Federal Government officers or employees acting as part of their official duties; on a worldwide, perpetual, irrevocable, royalty-free basis to BMJ Publishing Group Ltd ("BMJ") its licensees and where the relevant Journal is co-owned by BMJ to the co-owners of the Journal, to publish the Work in this journal and any other BMJ products and to exploit all rights, as set out in our licence.

The Submitting Author accepts and understands that any supply made under these terms is made by BMJ to the Submitting Author unless you are acting as an employee on behalf of your employer or a postgraduate student of an affiliated institution which is paying any applicable article publishing charge ("APC") for Open Access articles. Where the Submitting Author wishes to make the Work available on an Open Access basis (and intends to pay the relevant APC), the terms of reuse of such Open Access shall be governed by a Creative Commons licence – details of these licences and which Creative Commons licence will apply to this Work are set out in our licence referred to above.

Other than as permitted in any relevant BMJ Author's Self Archiving Policies, I confirm this Work has not been accepted for publication elsewhere, is not being considered for publication elsewhere and does not duplicate material already published. I confirm all authors consent to publication of this Work and authorise the granting of this licence.

For peer review only

Mortality in individuals with Abdominal Aortic Aneurysm

Do depressive symptoms worsen the prognosis? A HUNT Study

Linn Å. Nyrønning[1,2] Rebecka Hultgren[3,4] Grethe Albrektsen[5,6], Erney Mattsson[1,2]

Malin Stenman[3,7]

[1]Department of Surgery, Vascular Surgery, St Olavs Hospital, Trondheim, Norway,

[2]Department of Circulation and Medical Imaging, NTNU, Trondheim, Norway,

[3]Department of Molecular Medicine and Surgery, Karolinska Institutet, Stockholm, Sweden,

[4]Department of Vascular Surgery, Karolinska University Hospital, Stockholm, Sweden,

[5]Department of Public Health and Nursing, NTNU, Trondheim, Norway,

[6]Department of clinical and molecular medicine, NTNU, Trondheim, Norway,

[7]Perioperative Medicine and Intensive Care Function, Karolinska University Hospital, Stockholm, Sweden

Corresponding author:

Malin Stenman

Perioperative Medicine and Intensive Care Function

Karolinska University Hospital / Karolinska Institutet

SE-171 76 Stockholm, Sweden

E-mail: Malin.Stenman@ki.se

Word count: 2931

For peer review only

ABSTRACT

Background Abdominal aortic aneurysm (AAA) is a potentially life-threatening disease. Depression is associated with increased mortality in individuals with cardiovascular diseases, but such association is sparsely studied for AAA. The aim of this study was to compare overall mortality and prognostic impact of depression, between individuals with and without AAA.

Methods Population-based prospective study including 36616 participants (52.1 % women) from the Trøndelag Health Study (HUNT) in Norway. A total of 9428 individuals died during a median follow-up of 10 years at ages 60-90 years. Depressive symptoms were defined by a Hospital Anxiety and Depression (HADS-D) score ≥ 8. Data on AAA diagnoses and death were obtained from medical records and national registers. Hazard ratios (HR) from Cox proportional hazard regression models are reported.

Results A total of 583 (1.6%) AAAs were identified, and 4832 (13.2%) reported depressive symptoms. The hazard of death was considerably higher in persons with versus without AAA (HR 2.66; 95% CI 2.39-2.97). There was no clear adverse effect of depressive symptoms in individuals with AAA (HR=1.15; 95% CI 0.88-1.51), whereas a weak increase in risk was seen in the general population (HR=1.23; 95%CI 1.17-1.30). However, younger AAA patients (< 70 years) reporting depressive symptoms appeared to have a particularly poor prognosis.

Conclusion AAA was associated with increased mortality even after adjustments for age and established risk factors. Depressive symptoms did not worsen the prognosis notably in individuals with AAA, but a tendency was seen in persons diagnosed with AAA younger than 70 years.

For peer review only

Strengths and limitations of this study

- This is the first study to investigate the association between depressive symptoms and mortality in patients with AAA compared to a general population

- This study has used prospective data from the long running, longitudinal and well characterized HUNT study in Norway.

- Adjustment were made for age, sex and known comorbidities. The negative prognostic impact of depression appeared to be similar in persons diagnosed with AAA and in the general population, although a trend towards a worse prognosis was seen in the youngest individuals with AAA.

- The study focused on the association between depressive symptoms and mortality in patients with AAA. Further studies are needed to explore the relation between depression, AAA and mortality.

For peer review only

INTRODUCTION

Abdominal aortic aneurysm (AAA) is a localized widening of the abdominal aorta that often is asymptomatic and undiagnosed.[1] The most feared complication of an AAA is rupture, a life-threatening bleeding associated with a 100 % mortality if untreated.[1] To prevent deaths from a ruptured AAA, an early diagnosis and treatment in due time is essential. AAA is in general a late onset disease commonly diagnosed in persons older than 65 years that is associated with an unfavorable co-morbidity pattern and high mortality rates. [2, 3] Screening of men aged 65 years or older seems to reduce the risk of deaths related to AAA by up to 30 to 40 %.[4-7] whereas the effect on all-cause mortality is less clear.[8] Nonetheless, mortality rates in patients that have undergone surgery for AAA have been found to be higher than for patients with other cardiovascular diseases (CVDs). [9, 10] Most individuals diagnosed with AAA, however, live with an asymptomatic aneurysm or undergo elective surgery, and eventually die from other causes.

Depression is common in patients with cardiovascular disease [11] and is independently associated with increased cardiovascular morbidity and mortality.[2, 12, 13] In two recent large prospective, population-based studies, depression was identified as an independent risk factor for AAA.[14, 15] AAA and depression have both been associated with low-grade inflammation.[16, 17] Hence, inflammation is potentially a common risk factor that may explain the observed association between AAA and depression. Alternatively, the inflammation process evolves on the pathway between depression and the risk of AAA. Some previous studies have suggested that inflammation may increase the risk of rupture by triggering a more rapid growth of the aneurysm.[18, 19] If depression influences the risk of AAA development, AAA patients suffering from depression may have higher mortality rates

For peer review only

compared to AAA patients without depression. An adverse effect of depression on mortality rates in patients with cardiovascular disease has been established [13, 16, 17, 20-22], but we are not aware of any previous studies that have examined this issue among individuals with AAA. Identification of factors that trigger aneurysm growth or increase rupture risk is important in order to identify subgroups that may benefit from closer follow-up in the health care system. Increased knowledge of the etiology behind this severe condition may add valuable evidence to preventing development of the disease.

The aim of the present study was to examine the association between AAA and risk of death, and to examine whether depressive symptoms increase all-cause mortality to a larger extent in AAA patients than in a general population of the same age.

METHODS

This is a prospective population-based cohort study, based on data obtained from the Norwegian Trøndelag Health Study (HUNT). The study has been approved by the HUNT Study board of directors and the Regional Ethics Committee (2014/175/REK12 Midt-Norge), and was conducted in accordance with the Helsinki Declaration. The study was performed following the STROBE checklist for cohort studies (supplemental material).

The HUNT Study

HUNT is a large and ongoing multiphase population-based health study of people aged 20 years or older conducted in the county of Nord-Trøndelag in central Norway.[23] The databank contains extensive information about a wide variety of demographics and health-

For peer review only

related topics, including cardiovascular diseases. Data on depressive symptom was introduced in the second HUNT survey (HUNT2).

Study population

The present study population includes 36616 individuals (52.1 % women) who participated in HUNT2 and/or HUNT3 and had available data in HUNT on depressive symptoms. Median age at the first participation in HUNT was 58.6 years (range 40.6-89.8 years). The unique personal identification number was used to link data on AAA diagnoses (ICD-9/10) and death with data from the HUNT study.

Patient and Public Involvement

This study has not involved any direct contact with patients or the public. The study has only used de-identified data from the HUNT study.

Primary outcome and follow-up period

The primary outcome in this study was all-cause mortality. The Norwegian Cause of Death Registry was used to acquire information on dates of death. Each individual was considered to be at risk of death from the age of 60 years, or from the date of first HUNT participation at a higher age, until date of death or, reaching the age of 90 years, or closing date of the study (December 31, 2014). The lower and upper age limits were defined due to few deaths before the age of 60 years and few individuals with AAA at risk above the age of 90 years. Median age at start of follow-up was 60 years (range 60.0- 89.8 years) and median follow-up time was 10 years (1.1 days to 20 years). At the end of the study period, the median age was 73 years

For peer review only

(range 60-90 years). Among the 36616 individuals included in the study (51.2 % women), 9428 (44.0 % women) died during follow-up.

Ascertainment of AAA

Information on the diagnosis of AAA in the period 1995 through 2014 was obtained from local hospital registers (in-hospital and outpatient). An indicator variable for whether or not a person had been diagnosed with AAA was defined according to codes 441.3-4 and I71.3-4 in the ninth and tenth editions of the International Classification of Diseases (ICD 9/10 codes). All AAA diagnoses were manually verified using patient charts.

Assessment of depressive symptoms

The Hospital Anxiety and Depression Scale (HADS) was used to assess the degree of depressive symptoms. HADS is a self-report questionnaire developed to evaluate patients in somatic care and aims to measure levels of anxiety and depression symptoms during the preceding week. The questionnaire consists of two separate subscales ranging from 0-21: the HADS Anxiety (HADS-A) and HADS Depression (HADS-D). Only the HADS-D score was used in the present study. A cut-off at ≥ 8 (HADS-D ≥8) gives a specificity of 0.79 and a sensitivity of 0.83 compared with a clinical evaluation of depression.[24, 25]

Demographic and clinical factors

Information on potential confounders was assessed through responses on self-reported questionnaires and clinical examinations. Sex and civil status (categorized as living alone or with other) were assessed through questionnaires. Hypertension (yes, no) was defined as systolic blood pressure > 140 mmHg, diastolic blood pressure > 90 mmHg (average of second

For peer review only

and third measurements) or use of antihypertensive medication (self-reported). Body mass index (BMI) was calculated as weight/height^2 (kg/m^2), and divided into four categories (<25, 25-30, 30-35, ≥35 kg/m^2). Coronary heart disease (CHD, yes, no) was defined as a composite variable of self-reported history of myocardial infarction or angina pectoris. Diabetes (yes, no), smoking status (never, past, current) were defined according to questionnaire responses.

For peer review only

Statistical analysis

Descriptive statistics at start of follow-up are presented as mean (standard deviation, SD), median (interquartile range), and/or as frequencies (%), according to nature of the data. Kaplan-Meier survival curves for AAA (yes, no), and for combined categories defined by the dichotomized HADS-D score (<8, ≥8) and the indicator variable for AAA (yes, no), are presented. The log-rank test was applied to compare the overall survival between the groups. Unadjusted and adjusted hazard ratios (HR) for death with 95 % confidence intervals (CI) were calculated as estimate of relative risk in a Cox proportional hazard (PH) regression model. An interaction term between HADS and AAA was included in the model to evaluate whether the prognostic impact of depressive symptoms differed between persons with and without AAA. HUNT participants without a diagnosis of AAA were considered representative for the general population. HR values for combined categories of AAA and HADS were also calculated. The analyses were adjusted for sex, BMI category, smoking, CHD, diabetes mellitus, civil status, hypertension and AAA. Individuals with missing values on HADS-D (12.6 %) or other covariates were excluded from the analyses (complete case analysis, n= 34464). The proportional hazard (PH) assumption for each covariate in the Cox PH regression model was visually evaluated in log-minus-log plots. Predicted survival plots based on an adjusted Cox PH regression model,

stratified for AAA and depressive symptoms, were constructed to allow for deviation from the PH assumption.

Time-dependent covariates

Attained age was used as time scale both in the Kaplan-Meier survival analyses and in the Cox PH regression model. Except for sex, all prognostic factors were treated as time-, i.e. age-dependent covariates, allowing a shift in exposure categories during follow-up for individuals who participated in two HUNT surveys. The covariate values at start of follow-up were defined according to values obtained at the HUNT participation closest in time before the age of 60 years or at the first HUNT participation at an older age. The indicator variable for AAA was 0 (not present) until date (age) of diagnosis (recoded to 1, i.e. present). The diagnosis of AAA could occur either before, after or between two HUNT participations, depending on age at diagnosis and age at participation in a HUNT survey. A total of 27 individuals (2 women) had been diagnosed with AAA at start of follow-up, whereas an additional 556 individuals (149 women) were diagnosed during follow-up.

All statistical analyses were performed using Stata (Version IC.16.1 Stata Corp, College Station, TX). A p value <0.05 was considered significant (2-sided tests).

For peer review only

RESULTS

The distribution of the demographic and clinical characteristics of the study population at start of follow-up is presented in Table 1. A total of 583 individuals (432 men, 151 women) had a diagnosed AAA, and 4 832 (13.2%) individuals reported depressive symptoms (HADS-D ≥ 8). The proportion with a HADS-D score ≥ 8 was higher in individuals with AAA than in the general population (Table 1).

All-cause mortality in individuals with AAA versus the general population

The Kaplan Meier survival curves for individuals with and without AAA are shown in Figure 1. Overall, the estimated proportion of survivors was significantly lower in individuals with AAA compared to the general population (log rank test, p-value < 0.001). The unadjusted hazard of death in individuals with AAA was more than three times higher than in individuals without AAA (Table 2). The increased risk was less pronounced in the adjusted analysis but remained more than twice as high in the AAA group compared to the general population (Table 2).

Prognostic impact of depression in individuals with AAA versus the general population

Depressive symptoms (HADS-D ≥ 8) were associated with slightly poorer survival both in individuals with and without AAA (Figure 2A). The Kaplan Meier curves indicated a more pronounced negative prognostic effect from depressive symptoms in AAA individuals younger than 70 years, whereas no such age-dependent effect was seen in the general population. This pattern was confirmed in the log-log-plot used to evaluate the proportional hazard assumption in the Cox PH regression model (Supplemental material, Figure S1).

For peer review only

In the unadjusted Cox PH regression analyses (Table 2), calculated under the assumption of proportional hazard during the entire age interval (60-90 years), the HR of death in persons with versus without depressive symptoms (HADS-D ≥8 vs. < 8) was similar in individuals with AAA and in the general population. Adjustments for sex, smoking, coronary heart disease, diabetes, BMI, hypertension, and civil status, weakened the association with depression, particularly in the AAA group (Table 2, Figure 2B). In terms of relative risk, the adjusted HR values indicated a slightly weaker negative prognostic impact of depressive symptoms in the AAA group than in the general population, but the difference was not statistically significant (p, test for interaction = 0.62).

For peer review only

Table 1. Demographic and clinical characteristics at start of follow-up, total and by AAA

Characteristics	Total population n (%)	AAA Yes n (%)	AAA No n (%)	P-value
**Total**	36616 (100)	583 (100)	36033 (100)	
**Sex**				<0.001
Men	17883 (48.8)	432 (74.1)	17451 (48.4)	
Women	18733 (51.2)	151 (25.9)	18582 (51.6)	
**Age at measured HUNT**				<0.001
<60	20843 (56.9)			
60-69	9550 (26.1)			
70-79	5254 (14.3)			
≥80	969 (2.4)			
**HADS-D**				<0.001
<8	31784 (86.3)	477 (81.8)	31307 (86.9)	
≥8	4832 (13.2)	106 (18.2)	4726 (13.1)	
**Smoking**				<0.001
Never	12890 (35.9)	53 (9.2)	12837 (36.4)	
Past	12738 (35.5)	192 (33.5)	12546 (35.5)	
Current	10260 (28.6)	12546 (35.5)	9931 (28.1)	
**BMI**				0.26
<25	11198 (30.8)	177 (30.7)	11021 (30.8)	
25-29	17349 (47.8)	280 (48.5)	17069 (47.7)	
30-35	6107 (16.8)	103 (17.8)	6004 (16.8)	
≥35	1679 (4.6)	17 (3.0)	1662 (4.7)	
**CHD**	3347 (9.2)	423 (27.4)	160 (8.9)	<0.001
**Hypertension**	21124 (57.7)	423 (72.5)	20701 (57.5)	<0.001
**Diabetes**	1746 (4.8)	23 (4.0)	1723 (4.8)	0.09
**Civil status**				0.35
Cohabiting	26650 (72.9)	443 (76.0)	26207 (72.8)	
Living alone	9913 (27.1)	140 (24.0)	9773 (27.2)	

AAA = Abdominal Aortic Aneurysm, HADS = Hospital Anxiety and Depression Scale, D = depression subscale, BMI = Body Mass Index,
Missing data among those with valid HADS-D (n = 36616): Smoking, n = 728 (2.6%) BMI, n = 283 (0.8%), coronary heart disease, n = 23 (0.06%), systolic and diastolic blood pressure, n = 154 (0.4%), hypertension, n = 9 (0.02%), diabetes, n = 84 (0.2%), civil status, n = 53 (0.14%). Missing data on HADS-D, n = 5259 (12.6%).

For peer review only

Table 2. HR of death for AAA (yes vs. no) and for depressive symptoms (HADS-D ≥8 vs <8) among individuals with and without a diagnosis of AAA, respectively*

AAA	HADS-D	No. of deaths	Unadjusted HR (95%CI)	Adjusted HR (95% CI)**
No		9078	1.00	1.00
Yes		350	3.39 (3.01-3.78)	2.66 (2.39-2.97)
**Subgroups**				
**No**	<8	7430	1.00	1.00
	≥8	1648	1.28 (0.98-1.69)	1.23 (1.17-1.30)
**Yes**	<8	283	1.00	1.00
	≥8	67	1.28 (1.21-1.36)	1.15 (0.88-1.51)
**P-value, interaction**			0.90	0.62

Depressive symptoms were defined as a HADS-D score ≥8.
AAA = Abdominal Aortic Aneurysm, HR = Hazard ratio, CI = Confidence Interval, HADS= Hospital Anxiety and Depression Scale.
*HR from multiple Cox PH regression model with attained age as time scale, adjusted for HADS-D (<8 vs. ≥8), AAA, sex, coronary heart disease, diabetes mellitus, body mass index - category, smoking, hypertension, civil status.
**Complete case analyses: Number of individuals, n= 34464.

For peer review only

DISCUSSION

In this population-based prospective study, all-cause mortality was almost three times higher in individuals with AAA compared with a general population of the same age, after adjustment for common prognostic factors. We found no evidence of a stronger adverse effect of depressive symptoms on mortality in the AAA group, although there was a tendency towards a more pronounced adverse effect of depressive symptoms in individuals diagnosed before the age of 70 years.

The present finding of a higher mortality in individuals with vs. without AAA are consistent with a recent study reporting substantially lower overall long-term survival in patients undergoing successful AAA repair as compared to age-and sex-adjusted mortality rates in the general population.[26] An unfavorable co-morbidity profile commonly seen in AAA patients, including other cardiovascular diseases, is probably a contributing factor to the high crude mortality rates observed in the AAA group in general.[27] In our study, the risk estimate was weakened but the relative risk remained high also after adjusting for factors associated with both AAA and increased mortality, such as coronary heart disease, diabetes and smoking. Previously reported mortality rates in AAA patients have most often been restricted to patients treated for AAA. [26, 28] In contrast, our study included both untreated and treated AAA patients. Furthermore, we also compared with the risk of death in individuals without AAA. Hence, few previous studies have reported mortality rates that are directly comparable, and our results therefore indicate the need to further explore this issue.

To our knowledge, no previous studies have examined whether the impact of depressive symptoms on mortality differ in patients with AAA compared to a general population. The

For peer review only - http://bmjopen.bmj.com/site/about/guidelines.xhtml

adverse prognostic impact from depressive symptoms in the AAA group in our study, albeit not significant in the adjusted analyses, is in line with previous studies reporting hazard ratios in the range 1.15 to 1.30 for the impact of depression in populations with other cardiovascular diseases. [12, 13, 27, 29] The lack of a significant association in our study probably relates to the low number of individuals with AAA and depressive symptoms, leading to imprecise risk estimates. Notably, neither of the previous studies compared the adverse effect of depression among CVD patients to the impact observed in the general population. In our study, there was no evidence suggesting that depressive symptoms had larger impact in individuals with than without AAA. On the contrary, the estimated relative risks even indicated a slightly weaker association in the AAA group than in the general population. However, we did observe a more pronounced adverse effect of depressive symptoms in individuals diagnosed with AAA before the age of 70 years, whereas no such age-dependent effect was seen in the general population. A high number of pack years (smoking) and unfavorable heredity patterns have been reported to be more common in young compared to older AAA patients.[30] It is possible that these factors may accelerate the disease development in young AAA patients. Smoking is a known risk factor also for growth and rupture of AAA.[31, 32] If these factors also correlate with depressive symptoms in younger individuals, they may act as confounders for the observed age-specific association with depressive symptoms in our study. We adjusted for smoking habits, whereas data on heritability was not available. However, the low number of young individuals with AAA reporting depressive symptoms precluded calculation of age-specific adjusted risk estimates.

The biological mechanisms behind the increased mortality rates in individuals with AAA are yet to be clarified. However, inflammation seems to be an important part of both the AAA

For peer review only

pathogenesis and growth,[33, 34] which may relate to impact from noxious stimuli such as cigarette smoke, low density lipoprotein cholesterol and hypertension.[35] With increasing age, competent repair cells are not always available, artery repair fails and the inflammatory state increases.[1] Previous studies have shown that depression is associated with low-grade chronic inflammation.[1, 13, 36] Moreover, a negative impact from inflammatory processes on mortality in patients with CVDs and concurrent depression has been suggested.[37] Our finding that depression might only worsen the prognosis in younger, but not in older AAA patients might be somewhat contradictory. On the other hand, it is possible that individuals who develop AAA at a younger age have faster aneurysm growth that is more susceptible to a growth-enhancing effect from depression, potentially mediated by inflammation.

Strengths and limitations

A major strength of our study is that the results are based on data from a large, population-based, prospective study. The HUNT population is a validated and well-defined homogenous cohort that represents the general population in Norway. Data on risk factors were assessed at the individual level for all study participants, enabling tight adjustment for age (1- year intervals, time scale in the survival analyses), sex and other potential confounders. The amount of missing data for the covariates were low. All AAA cases were verified based on a certified hospital diagnosis and dates of death were obtained from national registers with compulsory recordings. It is, however, likely that there was some underdiagnosis of AAA cases. The HUNT study does not include ultrasound examinations of the abdominal aorta. The observed 1.6% point prevalence of AAA in our study is, however, in line with the expected rate at the time.[38] Unless a potential underdiagnosis of AAA differ by value of HADS-D, the risk estimates will not be biased. Some limitations must be considered. Most data on covariates

For peer review only

were self-reported. Symptoms of depression were assessed through self-reported questionnaires and not by a psychiatric diagnostic interview. We had no information about antidepressant use. The low number of individuals with severe depressive symptoms in our study population could be a result from recruitment bias. Individuals with severe depression might not adhere to screening programs or attend population-based studies to the same extent as non-depressed individuals. However, such recruitment bias would likely bias our results towards the null hypothesis.

CONCLUSION

This study has revealed a considerably higher all-cause mortality in individuals with AAA compared to a general population. The presence of depressive symptoms did not affect the risk of death more in the AAA group than in the general population, but there was a tendency towards a worse prognosis in individuals diagnosed with AAA before the age of 70 years. Further studies are needed to explore the relation between depression, AAA and mortality.

Acknowledgements

The Trøndelag Health Study (The HUNT Study) is a collaboration between HUNT Research Centre, (Faculty of Medicine and Health Sciences, NTNU, Norwegian University of Science and Technology), Trøndelag County Council, Central Norway Regional Health Authority, and the Norwegian Institute of Public Health.

Contributorship

LN contributed to study conception and design, data and statistical analysis, interpretation of study findings and drafting and critical review of the manuscript. RH contributed to study

For peer review only

conception and design, interpretation of study findings and drafting and critical review of the manuscript.

GA contributed to data and statistical analysis, interpretation of study findings and critical review of the manuscript. EM contributed to study conception and design and critical review of the manuscript. MS contributed to study conception and design, interpretation of study findings and drafting and critical review of the manuscript.

Data availability statement

No data are available

The Trøndelag Health Study (HUNT) has invited persons aged 13 - 100 years to four surveys between 1984 and 2019. Comprehensive data from more than 140.000 persons having participated at least once and biological material from 78.000 persons are collected. The data are stored in HUNT databank and biological material in HUNT biobank. HUNT Research Centre has permission from the Norwegian Data Inspectorate to store and handle these data. The key identification in the data base is the personal identification number given to all Norwegians at birth or immigration, whilst de-identified data are sent to researchers upon approval of a research protocol by the Regional Ethical Committee and HUNT Research Centre. To protect participants' privacy, HUNT Research Centre aims to limit storage of data outside HUNT databank, and cannot deposit data in open repositories. HUNT databank has precise information on all data exported to different projects and are able to reproduce these on request. There are no restrictions regarding data export given approval of applications to HUNT Research Centre. For more information see: https://eur01.safelinks.protection.outlook.com/?url=http%3A%2F%2Fwww.ntnu.edu%2Fhunt%2Fdata&data=04%7C01%7Cmalin.stenman%40ki.se%7C1bc54c35ed5f4ce79e05

For peer review only

08d8a7616b52%7Cbff7eef1cf4b4f32be3da1dda043c05d%7C0%7C0%7C637443384157146991%7CUnknown%7CTWFpbGZsb3d8eyJWIjoiMC4wLjAwMDAiLCJQIjoiV2luMzIiLCJBTiI6Ik1haWwiLCJXVCI6Mn0%3D%7C1000&sdata=GwGqQpTG4u%2FoslrgO43q64XHIpvyeEMTSCo%2ByrTn7i4%3D&reserved=0

Competing interests

None declared

Funding

This research has been supported by the Swedish Heart-Lung Foundation (Hultgren) grant number: 20190553 and 20180506.

Patient consent for publication

Not required

Disclosures

The authors of this manuscript have no potential conflicts of interest.

For peer review only

REFERENCES

1. Sakalihasan N, Michel JB, Katsargyris A, et al. Abdominal aortic aneurysms. *Nat Rev Dis Primers* 2018;4:34.
2. Hare DL, Toukhsati SR, Johansson P, et al. Depression and cardiovascular disease: a clinical review. *Eur Heart J* 2014;35:1365-72.
3. Bath MF, Sidloff D, Saratzis A, et al. Impact of abdominal aortic aneurysm screening on quality of life. *Br J Surg* 2018;105:203-08.
4. Wanhainen A, Hultgren R, Linne A, et al. Outcome of the Swedish Nationwide Abdominal Aortic Aneurysm Screening Program. *Circulation* 2016;134:1141-48.
5. Norman PE, Jamrozik K, Lawrence-Brown MM, et al. Population based randomised controlled trial on impact of screening on mortality from abdominal aortic aneurysm. *Bmj* 2004;329:1259.
6. Ashton HA, Buxton MJ, Day NE, et al. The Multicentre Aneurysm Screening Study (MASS) into the effect of abdominal aortic aneurysm screening on mortality in men: a randomised controlled trial. *Lancet* 2002;360:1531-9.
7. Lindholt JS, Juul S, Fasting H, et al. Screening for abdominal aortic aneurysms: single centre randomised controlled trial. *Bmj* 2005;330:750.
8. Ali MU, Fitzpatrick-Lewis D, Miller J, et al. Screening for abdominal aortic aneurysm in asymptomatic adults. *J Vasc Surg* 2016;64:1855-68.
9. Timmers TK, van Herwaarden JA, de Borst GJ, et al. Long-term survival and quality of life after open abdominal aortic aneurysm repair. *World J Surg* 2013;37:2957-64.
10. de Bruin JL, Baas AF, Heymans MW, et al. Statin therapy is associated with improved survival after endovascular and open aneurysm repair. *J Vasc Surg* 2014;59:39-44.e1.
11. Harshfield EL, Pennells L, Schwartz JE, et al. Association Between Depressive Symptoms and Incident Cardiovascular Diseases. *Jama* 2020;324:2396-405.
12. Stenman M, Holzmann MJ, Sartipy U. Relation of major depression to survival after coronary artery bypass grafting. *Am J Cardiol* 2014;114:698-703.
13. Nemeroff CB, Goldschmidt-Clermont PJ. Heartache and heartbreak--the link between depression and cardiovascular disease. *Nat Rev Cardiol* 2012;9:526-39.
14. Daskalopoulou M, George J, Walters K, et al. Depression as a Risk Factor for the Initial Presentation of Twelve Cardiac, Cerebrovascular, and Peripheral Arterial Diseases: Data Linkage Study of 1.9 Million Women and Men. *PLoS One* 2016;11:e0153838.
15. Nyronning LA, Stenman M, Hultgren R, et al. Symptoms of Depression and Risk of Abdominal Aortic Aneurysm: A HUNT Study. *J Am Heart Assoc* 2019;8:e012535.
16. Pan A, Sun Q, Okereke OI, et al. Depression and risk of stroke morbidity and mortality: a meta-analysis and systematic review. *Jama* 2011;306:1241-9.
17. Mykletun A, Bjerkeset O, Overland S, et al. Levels of anxiety and depression as predictors of mortality: the HUNT study. *Br J Psychiatry* 2009;195:118-25.
18. Sakalihasan N, Van Damme H, Gomez P, et al. Positron emission tomography (PET) evaluation of abdominal aortic aneurysm (AAA). *Eur J Vasc Endovasc Surg* 2002;23:431-6.
19. Dale MA, Ruhlman MK, Baxter BT. Inflammatory cell phenotypes in AAAs: their role and potential as targets for therapy. *Arterioscler Thromb Vasc Biol* 2015;35:1746-55.
20. Almeida OP, Ford AH, Hankey GJ, et al. Depression, antidepressants and the risk of cardiovascular events and death in older men. *Maturitas* 2019;128:4-9.

For peer review only

21. Cuijpers P, Vogelzangs N, Twisk J, et al. Comprehensive meta-analysis of excess mortality in depression in the general community versus patients with specific illnesses. *Am J Psychiatry* 2014;171:453-62.
22. Letnes JM, Torske MO, Hilt B, et al. Symptoms of depression and all-cause mortality in farmers, a cohort study: the HUNT study, Norway. *BMJ Open* 2016;6:e010783.
23. Krokstad S, Langhammer A, Hveem K, et al. Cohort Profile: the HUNT Study, Norway. *Int J Epidemiol* 2013;42:968-77.
24. Bjelland I, Dahl AA, Haug TT, et al. The validity of the Hospital Anxiety and Depression Scale. An updated literature review. *J Psychosom Res* 2002;52:69-77.
25. Myrtveit SM, Ariansen AM, Wilhelmsen I, et al. A population based validation study of self-reported pensions and benefits: the Nord-Trondelag health study (HUNT). *BMC Res Notes* 2013;6:27.
26. Bulder RMA, Talvitie M, Bastiaannet E, et al. Long-term Prognosis After Elective AAA Repair is Poor in Women and Men: The Challenges Remain. *Ann Surg* 2020.
27. Toghill BJ, Saratzis A, Bown MJ. Abdominal aortic aneurysm-an independent disease to atherosclerosis? *Cardiovasc Pathol* 2017;27:71-75.
28. Norman PE, Semmens JB, Lawrence-Brown MM, et al. Long term relative survival after surgery for abdominal aortic aneurysm in western Australia: population based study. *Bmj* 1998;317:852-6.
29. Rajan S, McKee M, Rangarajan S, et al. Association of Symptoms of Depression With Cardiovascular Disease and Mortality in Low-, Middle-, and High-Income Countries. *JAMA Psychiatry* 2020;77:1052-63.
30. Howard DP, Banerjee A, Fairhead JF, et al. Age-specific incidence, risk factors and outcome of acute abdominal aortic aneurysms in a defined population. *Br J Surg* 2015;102:907-15.
31. Stackelberg O, Wolk A, Eliasson K, et al. Lifestyle and Risk of Screening-Detected Abdominal Aortic Aneurysm in Men. *J Am Heart Assoc* 2017;6.
32. Stackelberg O, Björck M, Larsson SC, et al. Sex differences in the association between smoking and abdominal aortic aneurysm. *Br J Surg* 2014;101:1230-7.
33. Kuivaniemi H, Ryer EJ, Elmore JR, et al. Understanding the pathogenesis of abdominal aortic aneurysms. *Expert Rev Cardiovasc Ther* 2015;13:975-87.
34. Eagleton MJ. Inflammation in abdominal aortic aneurysms: cellular infiltrate and cytokine profiles. *Vascular* 2012;20:278-83.
35. Rocha VZ, Libby P. Obesity, inflammation, and atherosclerosis. *Nature reviews Cardiology* 2009;6:399-409.
36. Raedler TJ. Inflammatory mechanisms in major depressive disorder. *Curr Opin Psychiatry* 2011;24:519-25.
37. Celano CM, Huffman JC. Depression and cardiac disease: a review. *Cardiol Rev* 2011;19:130-42.
38. Ulug P, Powell JT, Sweeting MJ, et al. Meta-analysis of the current prevalence of screen-detected abdominal aortic aneurysm in women. *Br J Surg* 2016;103:1097-104.

For peer review only

Figure legends

Figure 1. The Kaplan-Meier curve of all-cause mortality and numbers at risk during follow-up according to AAA (no, yes)

Figure 2. Expected proportion of survivors in combined categories of AAA (no, yes) and HADS-D (<8 versus ≥8), based on A) Kaplan-Meier approach, and B) a stratified Cox PH regression model, adjusted.
AAA = Abdominal Aortic Aneurysm, HADS = Hospital Anxiety and Depression Scale.

For peer review only

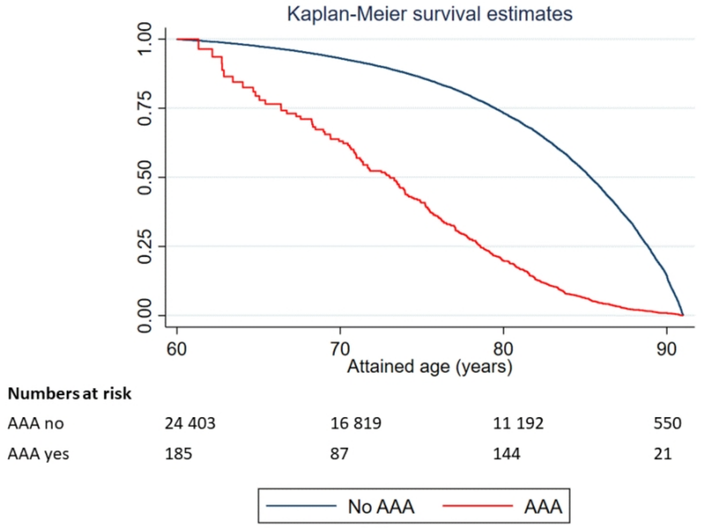

451x254mm (72 x 72 DPI)

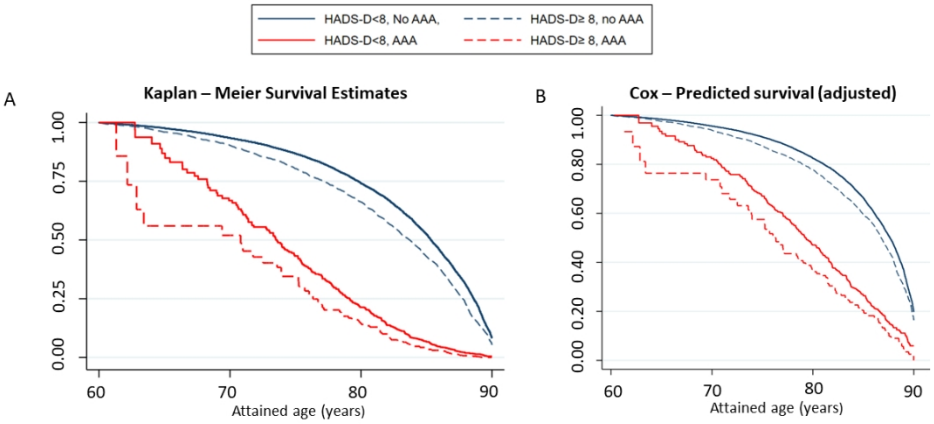

Numbers at risk

HADS-D < 8, No AAA	19 931	13 690	8 129	320
HADS-D ≥ 8, No AAA	2 559	1 986	1 462	71
HADS-D < 8, AAA	138	68	107	10
HADS-D ≥ 8, AAA	45	10	23	5

Supplemental material

Mortality in individuals with Abdominal Aortic Aneurysm

Do depressive symptoms worsen the prognosis? A HUNT Study

Linn Å. Nyrønning[1,2] Rebecka Hultgren[3,4] Grethe Albrektsen[5,6], Erney Mattsson[1,2]

Malin Stenman[3,7]

[1]Department of Surgery, Vascular Surgery, St Olavs Hospital, Trondheim, Norway,

[2]Department of Circulation and Medical Imaging, NTNU, Trondheim, Norway,

[3]Department of Molecular Medicine and Surgery, Karolinska Institutet, Stockholm, Sweden,

[4]Department of Vascular Surgery, Karolinska University Hospital, Stockholm, Sweden,

[5]Department of Public Health and Nursing, NTNU, Trondheim, Norway,

[6]Department of clinical and molecular medicine, NTNU, Trondheim, Norway,

[7]Perioperative Medicine and Intensive Care Function, Karolinska University Hospital, Stockholm, Sweden

Corresponding author:

Malin Stenman

Perioperative Medicine and Intensive Care Function

Karolinska University Hospital / Karolinska Institutet

SE-171 76 Stockholm, Sweden

E-mail: Malin.Stenman@ki.se

For peer review only

Figure S1. “Log - log plot” to test the proportional hazards assumption. If the plotted lines are reasonably parallel, the proportional hazards assumption has not been violated.

For peer review only

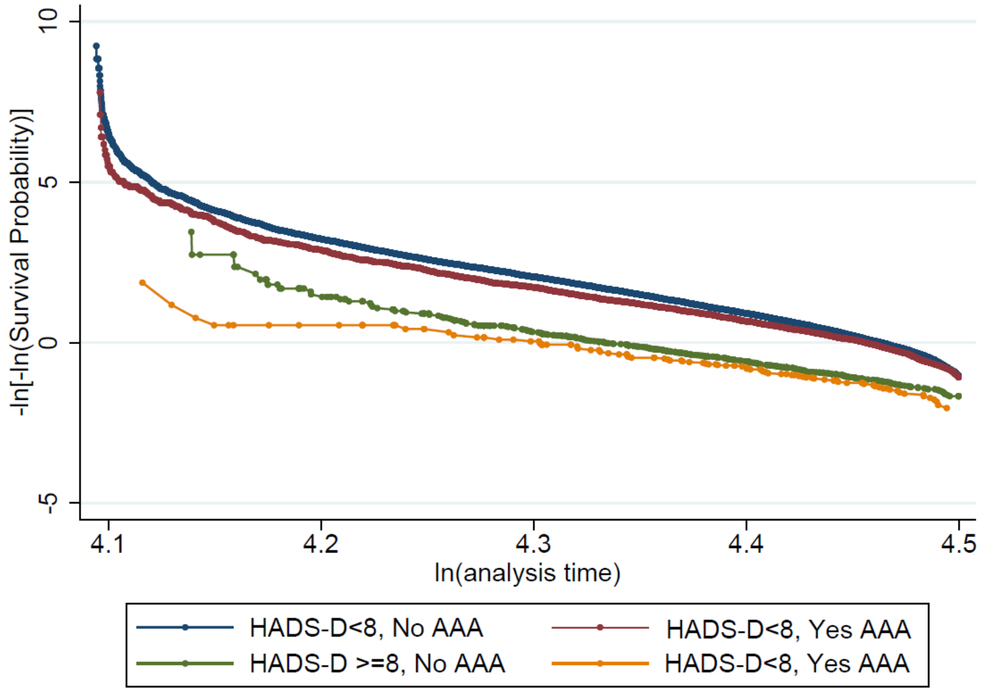
10
5
0
-5
-ln[-ln(Survival Probability)]
4.1
4.2
4.3
4.4
4.5
ln(analysis time)
HADS-D<8, No AAA
HADS-D<8, Yes AAA
HADS-D >=8, No AAA
HADS-D<8, Yes AAA

STROBE Statement—Checklist of items that should be included in reports of ***cohort studies***

	Item No	Recommendation
**Title and abstract**	1	(*a*) Indicate the study's design with a commonly used term in the title or the abstract
		(*b*) Provide in the abstract an informative and balanced summary of what was done and what was found
**Introduction**		
Background/rationale	2	Explain the scientific background and rationale for the investigation being reported
Objectives	3	State specific objectives, including any prespecified hypotheses
**Methods**		
Study design	4	Present key elements of study design early in the paper
Setting	5	Describe the setting, locations, and relevant dates, including periods of recruitment, exposure, follow-up, and data collection
Participants	6	(*a*) Give the eligibility criteria, and the sources and methods of selection of participants. Describe methods of follow-up
		(*b*) For matched studies, give matching criteria and number of exposed and unexposed
Variables	7	Clearly define all outcomes, exposures, predictors, potential confounders, and effect modifiers. Give diagnostic criteria, if applicable
Data sources/ measurement	8*	For each variable of interest, give sources of data and details of methods of assessment (measurement). Describe comparability of assessment methods if there is more than one group
Bias	9	Describe any efforts to address potential sources of bias
Study size	10	Explain how the study size was arrived at
Quantitative variables	11	Explain how quantitative variables were handled in the analyses. If applicable, describe which groupings were chosen and why
Statistical methods	12	(*a*) Describe all statistical methods, including those used to control for confounding
		(*b*) Describe any methods used to examine subgroups and interactions
		(*c*) Explain how missing data were addressed
		(*d*) If applicable, explain how loss to follow-up was addressed
		(*e*) Describe any sensitivity analyses
**Results**		
Participants	13*	(a) Report numbers of individuals at each stage of study—eg numbers potentially eligible, examined for eligibility, confirmed eligible, included in the study, completing follow-up, and analysed
		(b) Give reasons for non-participation at each stage
		(c) Consider use of a flow diagram
Descriptive data	14*	(a) Give characteristics of study participants (eg demographic, clinical, social) and information on exposures and potential confounders
		(b) Indicate number of participants with missing data for each variable of interest
		(c) Summarise follow-up time (eg, average and total amount)
Outcome data	15*	Report numbers of outcome events or summary measures over time
Main results	16	(*a*) Give unadjusted estimates and, if applicable, confounder-adjusted estimates and their precision (eg, 95% confidence interval). Make clear which confounders were adjusted for and why they were included
		(*b*) Report category boundaries when continuous variables were categorized
		(*c*) If relevant, consider translating estimates of relative risk into absolute risk for a meaningful time period

For peer review only

For peer review only - http://bmjopen.bmj.com/site/about/guidelines.xhtml

Other analyses	17	Report other analyses done—eg analyses of subgroups and interactions, and sensitivity analyses
**Discussion**		
Key results	18	Summarise key results with reference to study objectives
Limitations	19	Discuss limitations of the study, taking into account sources of potential bias or imprecision. Discuss both direction and magnitude of any potential bias
Interpretation	20	Give a cautious overall interpretation of results considering objectives, limitations, multiplicity of analyses, results from similar studies, and other relevant evidence
Generalisability	21	Discuss the generalisability (external validity) of the study results
**Other information**		
Funding	22	Give the source of funding and the role of the funders for the present study and, if applicable, for the original study on which the present article is based

*Give information separately for exposed and unexposed groups.

Note: An Explanation and Elaboration article discusses each checklist item and gives methodological background and published examples of transparent reporting. The STROBE checklist is best used in conjunction with this article (freely available on the Web sites of PLoS Medicine at http://www.plosmedicine.org/, Annals of Internal Medicine at http://www.annals.org/, and Epidemiology at http://www.epidem.com/). Information on the STROBE Initiative is available at http://www.strobe-statement.org.

For peer review only

The prognostic impact of depressive symptoms on all-cause mortality in individuals with abdominal aortic aneurysm compared to the general population. A population-based prospective HUNT study in Norway

Journal:	*BMJ Open*
Manuscript ID	bmjopen-2021-049055.R1
Article Type:	Original research
Date Submitted by the Author:	19-May-2021
Complete List of Authors:	Nyrønning, Linn Å.; Department of Surgery, Vascular Surgery; NTNU Department of Circulation and Medical Imaging<br>Hultgren, Rebecka; Karolinska Institute, Deparment of molecular medicine and surgery; Karolinska Universitetssjukhuset, Department of Vascular Surgery<br>Albrektsen, Grethe; Norges teknisk-naturvitenskapelige universitet, Department of public health and general practice<br>Mattsson, Erney; Department of Surgery, Vascular Surgery; NTNU Department of Circulation and Medical Imaging<br>Stenman, Malin ; Karolinska Institutet, Department of Molecular Medicine and Surgery; Karolinska University Hospital, Department of Molecular Medicine and Surgery
**Primary Subject Heading**:	Epidemiology
Secondary Subject Heading:	Epidemiology, Cardiovascular medicine
Keywords:	EPIDEMIOLOGY, Vascular medicine < INTERNAL MEDICINE, Vascular surgery < SURGERY, VASCULAR SURGERY

I, the Submitting Author has the right to grant and does grant on behalf of all authors of the Work (as defined in the below author licence), an exclusive licence and/or a non-exclusive licence for contributions from authors who are: i) UK Crown employees; ii) where BMJ has agreed a CC-BY licence shall apply, and/or iii) in accordance with the terms applicable for US Federal Government officers or employees acting as part of their official duties; on a worldwide, perpetual, irrevocable, royalty-free basis to BMJ Publishing Group Ltd ("BMJ") its licensees and where the relevant Journal is co-owned by BMJ to the co-owners of the Journal, to publish the Work in this journal and any other BMJ products and to exploit all rights, as set out in our licence.

The Submitting Author accepts and understands that any supply made under these terms is made by BMJ to the Submitting Author unless you are acting as an employee on behalf of your employer or a postgraduate student of an affiliated institution which is paying any applicable article publishing charge ("APC") for Open Access articles. Where the Submitting Author wishes to make the Work available on an Open Access basis (and intends to pay the relevant APC), the terms of reuse of such Open Access shall be governed by a Creative Commons licence – details of these licences and which Creative Commons licence will apply to this Work are set out in our licence referred to above.

Other than as permitted in any relevant BMJ Author's Self Archiving Policies, I confirm this Work has not been accepted for publication elsewhere, is not being considered for publication elsewhere and does not duplicate material already published. I confirm all authors consent to publication of this Work and authorise the granting of this licence.

For peer review only

The prognostic impact of depressive symptoms on all-cause mortality in individuals with abdominal aortic aneurysm compared to the general population. A population-based prospective HUNT study in Norway

Linn Å. Nyrønning[1,2] Rebecka Hultgren[3,4] Grethe Albrektsen[5,6], Erney Mattsson[1,2] Malin Stenman[3,7]

[1]Department of Surgery, Vascular Surgery, St Olavs Hospital, Trondheim, Norway,

[2]Department of Circulation and Medical Imaging, NTNU, Trondheim, Norway,

[3]Department of Molecular Medicine and Surgery, Karolinska Institutet, Stockholm, Sweden,

[4]Department of Vascular Surgery, Karolinska University Hospital, Stockholm, Sweden,

[5]Department of Public Health and Nursing, NTNU, Trondheim, Norway,

[6]Department of clinical and molecular medicine, NTNU, Trondheim, Norway,

[7]Perioperative Medicine and Intensive Care Function, Karolinska University Hospital, Stockholm, Sweden

Corresponding author:

Malin Stenman

Perioperative Medicine and Intensive Care Function

Karolinska University Hospital / Karolinska Institutet

SE-171 76 Stockholm, Sweden

E-mail: Malin.Stenman@ki.se

Word count: 3325

For peer review only

ABSTRACT

Background Abdominal aortic aneurysm (AAA) is a potentially life-threatening disease but the high mortality rate is linked to high age and comorbidity pattern. Depression is associated with increased mortality in the general population and individuals with cardiovascular diseases, but this is sparsely studied for AAA. The aim was to examine the prognostic impact of depressive symptoms on all-cause mortality in individuals with AAA and compare with a general population of the same age and risk profile.

Methods Population-based prospective study including 36616 participants (52.1 % women) from the Trøndelag Health Study (HUNT) in Norway. A total of 9428 individuals died during a median follow-up of 10 years at ages 60-90 years. Depressive symptoms were defined by a Hospital Anxiety and Depression (HADS-D) score ≥ 8. Data on AAA diagnoses and death were obtained from medical records and national registers. Hazard ratios (HR) from Cox proportional hazard regression models are reported.

Results A total of 4832 (13.2%) individuals reported depressive symptoms, whereas 583 (1.6%) AAAs were identified. The adjusted hazard of death was 2.66 times higher in persons with AAA compared to the general population (95% CI 2.39-2.97). There was no adverse effect of depressive symptoms in individuals with AAA (HR=1.15;95% CI 0.88-1.51), whereas an increased risk was seen in the general population (HR=1.23;95%CI 1.17-1.30).

Conclusion The overall risk of death was considerably higher in individuals with AAA compared to a general population of the same age and risk profile. Depressive symptoms did not influence mortality in the AAA group.

For peer review only - http://bmjopen.bmj.com/site/about/guidelines.xhtml

Strengths and limitations of this study

- The results from this study are based on data from a large and population-based prospective study in Norway.
- The general population served as comparison group when evaluating the prognostic impact of depressive symptoms on all-cause mortality in individuals diagnosed with abdominal aortic aneurysm.
- The risk estimates were adjusted for potential confounders, such as age, sex, coronary heart disease, diabetes, BMI, hypertension and civil status.
- All AAA cases were verified based on a certified hospital diagnosis, but due to the lack of ultrasound measurement in HUNT, the prevalence of AAA in the general population may have been underestimated.
- Data on depressive symptoms and comorbidity patterns were self-reported.
- Use of time-dependent variables made it possible to update the risk profiles during follow-up, but not all individuals participated in HUNT more than once.

For peer review only - http://bmjopen.bmj.com/site/about/guidelines.xhtml

INTRODUCTION

Abdominal aortic aneurysm (AAA) is a localized widening of the abdominal aorta that often is asymptomatic and undiagnosed.[1] The most feared complication of an AAA is rupture, a life-threatening bleeding associated with fatal outcome if untreated.[1] To prevent deaths from a ruptured AAA, an early diagnosis and treatment in due time is essential. AAA is in general a late onset disease, that is associated with an unfavorable co-morbidity pattern and high mortality rates. [2, 3] Screening of men aged 65 years or older seems to reduce the risk of deaths related to AAA by up to 30 to 40 %.[4-7] whereas the effect on all-cause mortality is less clear.[8] Nonetheless, mortality rates in patients that have undergone surgery for AAA have been found to be higher than for patients with other cardiovascular diseases (CVDs). [9, 10] High mortality in patients diagnosed with AAA has been linked to high age and unfavorable comorbidity patters, including death from cardiovascular diseases and other smoking-related diseases. Many individuals diagnosed with AAA live with an asymptomatic aneurysm or undergo elective surgery, and eventually die from other causes.[11] Very few previous studies have compared the overall mortality in individuals diagnosed with AAA to a general population of the same age and with the same risk profile.

Depression is associated with increased mortality in the general population.[12] Moreover, depression is common in patients with cardiovascular disease [13] and is independently associated with increased cardiovascular morbidity and mortality.[2, 14, 15] In two recent large prospective, population-based studies, depression was associated with increased risk of AAA.[16, 17] AAA and depression have both been associated with low-grade inflammation.[12, 18] Hence, inflammation could be a common risk factor that may explain the observed association between AAA and depression. Alternatively, the inflammation

For peer review only

process evolves on the pathway between depression and the risk of AAA. Previous studies have suggested that inflammation may increase the risk of rupture by triggering a more rapid growth of the aneurysm.[19, 20] If depression, as a proxy for low grade inflammation, influences both the risk and progression of AAA development, an adverse prognostic impact of depression may be particularly pronounced in individuals with AAA. An adverse effect of depression on mortality rates in patients with other cardiovascular diseases has been established [12, 15, 18, 21-23], but we are not aware of any previous studies that have examined the prognostic impact of depression in individuals with AAA compared to a general population.

The main aim of the present study was to examine whether the prognostic impact of depressive symptoms on all-cause mortality in individuals diagnosed with AAA differed from the established adverse effect in the general population. Moreover, we investigate whether there was an overall difference in all-cause mortality between individuals diagnosed with AAA and the general population of the same age and with the same risk profile.

METHODS

This is a prospective population-based cohort study, based on data obtained from the Norwegian Trøndelag Health Study (HUNT). The study has been approved by the HUNT Study board of directors and the Regional Ethics Committee (2014/175/REK12 Midt-Norge), and was conducted in accordance with the Helsinki Declaration. The study was performed following the STROBE checklist for cohort studies (supplemental material).

For peer review only

The HUNT Study

HUNT is a large and ongoing multiphase population-based health study of people aged 20 years or older conducted in the county of Nord-Trøndelag in central Norway.[24] The databank contains extensive information about a wide variety of demographics and health-related topics. Data on depressive symptom was introduced in the second HUNT survey (HUNT2).

Study population

The present study population includes 36616 individuals (52.1 % women) who participated in HUNT2 and/or HUNT3 and had available data in HUNT on depressive symptoms. Median age at the first participation in HUNT was 58.6 years (range 40.6-89.8 years). The unique personal identification number was used to link data on AAA diagnoses (ICD-9/10) and death with data from the HUNT study.

Patient and Public Involvement

This study has no direct contact with patients or the public. The study utilize de-identified data from the HUNT study.

Primary outcome and follow-up period

The primary outcome in this study was all-cause mortality. The Norwegian Cause of Death Registry was used to acquire information on dates of death. Each individual was considered to be at risk of death from the age of 60 years, or from the date of first HUNT participation at

For peer review only - http://bmjopen.bmj.com/site/about/guidelines.xhtml

a higher age, until date of death or, reaching the age of 90 years, or closing date of the study (December 31, 2014). The lower and upper age limits were defined due to few deaths before the age of 60 years and few individuals with AAA at risk above the age of 90 years. Median age at start of follow-up was 60 years (range 60.0- 89.8 years) and median follow-up time was 10 years (1.1 days to 20 years). At the end of the study period, the median age was 73 years (range 60-90 years). Among the 36616 individuals included in the study (51.2 % women), 9428 (44.0 % women) died during follow-up.

Ascertainment of AAA

Information on the diagnosis of AAA in the period 1995 through 2014 was obtained from local hospital registers (in-hospital and outpatient). An indicator variable for whether or not a person had been diagnosed with AAA was defined according to codes 441.3-4 and I71.3-4 in the ninth and tenth editions of the International Classification of Diseases (ICD 9/10 codes). All AAA diagnoses were manually verified using patient charts.

Assessment of depressive symptoms

The Hospital Anxiety and Depression Scale (HADS) was used to assess the degree of depressive symptoms. HADS is a self-report questionnaire developed to evaluate patients in somatic care and aims to measure levels of anxiety and depression symptoms during the preceding week. The questionnaire consists of two separate subscales ranging from 0-21: the HADS Anxiety (HADS-A) and HADS Depression (HADS-D). Only the HADS-D score was used in the present study. A HADS-D score ≥ 8 was defined as having depressive symptoms. A cut-off at ≥ 8 (HADS-D ≥8) gives a specificity of 0.79 and a sensitivity of 0.83 compared with a clinical evaluation of depression.[25, 26]

For peer review only - http://bmjopen.bmj.com/site/about/guidelines.xhtml

Demographic and clinical factors

Information on potential confounders was assessed through responses on self-reported questionnaires and clinical examinations. Sex and civil status (categorized as living alone or with other) were assessed through questionnaires. Hypertension (yes, no) was defined as systolic blood pressure > 140 mmHg, diastolic blood pressure > 90 mmHg (average of second and third measurements) or use of antihypertensive medication (self-reported). Body mass index (BMI) was calculated as weight/height^2 (kg/m^2), and divided into four categories (<25, 25-30, 30-35, ≥35 kg/m^2). Coronary heart disease (CHD, yes, no) was defined as a composite variable of self-reported history of myocardial infarction or angina pectoris. Diabetes (yes, no), smoking status (never, past, current) were defined according to questionnaire responses.

Statistical analysis

Descriptive statistics at start of follow-up are presented as mean (standard deviation, SD), median (interquartile range), and/or as frequencies (%), according to nature of the data. The Kaplan-Meier approach was applied to calculate the expected proportion of non-survivors (proportion of deaths) at different ages for AAA (yes, no), and for combined categories of the HADS-D score (<8, ≥8) and the indicator variable for AAA (yes, no). The log-rank test was applied to compare the overall survival between the groups. Unadjusted and adjusted hazard ratios (HR) for death with 95 % confidence intervals (CI) were calculated as estimate of relative risk in a Cox proportional hazard (PH) regression model. An interaction term between HADS-D and AAA (multiplicative model, using relative risk as measure of effect) was included in the Cox PH regression model to evaluate whether the prognostic impact of depressive symptoms

For peer review only - http://bmjopen.bmj.com/site/about/guidelines.xhtml

differed between persons with and without AAA. HUNT participants without a diagnosis of AAA were considered representative for the general population. The analyses were adjusted for age (time-scale), sex, BMI category, smoking, CHD, diabetes mellitus, hypertension and civil status. In the analyses of the overall association between AAA and mortality, we also adjusted for depressive symptoms. A backward stepwise procedure was applied to evaluate the individual contributions from single factors and to search for signs of potential overfitting of the model. The proportional hazard (PH) assumption for each covariate in the Cox PH regression model was visually evaluated in log-minus-log plots. Predicted survival plots based on the Cox PH regression model (unadjusted, adjusted) are also presented. Individuals with missing values on HADS-D (12.6 %) or other covariates were excluded from the analyses (complete case analysis, n= 34 464).

Time-varying covariates

Attained age was used as time scale both in the Kaplan-Meier survival analyses and in the Cox PH regression model. Except for sex, all prognostic factors were treated as time-varying covariates, allowing a shift in exposure categories with increasing age for individuals who participated in two HUNT surveys. The covariate values at start of follow-up were defined according to values obtained at the HUNT participation closest in time before the age of 60 years or at the first HUNT participation at an older age. The indicator variable for AAA was 0 (not present) until date (age) of diagnosis (recoded to 1, i.e. present). The diagnosis of AAA could occur either before, after or between two HUNT participations, depending on age at diagnosis and age at participation in a HUNT survey. A total of 27 individuals (2 women) had been diagnosed with AAA at start of follow-up from the age of 60 years, whereas an additional 556 individuals (149 women) were diagnosed during follow-up.

For peer review only - http://bmjopen.bmj.com/site/about/guidelines.xhtml

All statistical analyses were performed using Stata (Version IC.16.1 Stata Corp, College Station, TX). A p value <0.05 was considered significant (2-sided tests).

For peer review only

For peer review only - http://bmjopen.bmj.com/site/about/guidelines.xhtml

RESULTS

The distribution of the demographic and clinical characteristics of the study population at start of follow-up is presented in Table 1. A total of 583 individuals (432 men, 151 women) had a diagnosed AAA, and 4 832 (13.2%) individuals reported depressive symptoms (HADS-D ≥ 8). The proportion with a HADS-D score ≥ 8 was higher in individuals with than without a diagnosed AAA (Table 1). Compared with persons without AAA (considered representative for the general population), individuals with AAA were more often men (74.1 vs. 48.4 %), current smokers (35.5 vs. 57.5 %) and reported more coronary heart disease (27.4 vs. 8.9 %) and hypertension (72.5 vs. 57.5 %). Moreover, a larger proportion in the AAA group was obese (4.7 vs 3.0 %), but no significant association with BMI category was found.

All-cause mortality in individuals with AAA versus the general population

A Kaplan Meier failure plot of the failure function, with expected proportion of deaths at different ages, for individuals with and without a diagnosed AAA, is shown in Figure 1A. Individuals with AAA died at a younger age compared to the general population (median age at death 73.0 vs. 82.5 years, (log rank test, p-value < 0.001). The unadjusted hazard of death in individuals with AAA was more than three times the hazard of death in the general population (Table 2, HR 3.39, 95 % CI 3.01-3.78). The difference was less pronounced in the adjusted analysis but the hazard remained more than twice as high in the AAA group (Table 2, HR 2.66, 95 % CI 2.39-2.97).

Prognostic impact of depression in individuals with AAA versus the general population

The expected proportions of deaths by attained age in the four subgroups defined by AAA (no, yes) and HADS-D (<8, ≥8), are shown in Figure 1 B. Median age at death in individuals with and

For peer review only - http://bmjopen.bmj.com/site/about/guidelines.xhtml

without depressive symptoms in the AAA group was 70.8 and 73.5 years, whereas the corresponding numbers in the general population were 83.8 and 85.4 years.

In the unadjusted Cox PH regression analyses, the adverse effect of depressive symptoms in individuals with AAA was rather similar to the association seen in the general population (Table 2, HR for HADS-D ≥8 vs. < 8 of 1.28 in both groups). In the analyses adjusted for sex, smoking, coronary heart disease, diabetes, BMI, hypertension, and civil status (Table 2) there was no significant prognostic impact if depressive symptoms in individuals with AAA (HR 1.15, 95%CI 0.88-1.51), whereas a weak adverse effect remained in the general population (HR 1.23, 95%CI 1.17-1.30). The difference in the prognostic impact of depressive symptoms between the two groups did not reach statistical significance (p, test for interaction = 0.62). The factors contributing most to the reduced strength of the association with depressive symptoms were coronary heart disease, BMI and smoking. There was no single factor that explained the flattening of the risk estimate, or led to very imprecise estimates.

Age-specific effects

The unadjusted Kaplan Meier plots (Figure 1B) indicated a more pronounced negative prognostic effect from depressive symptoms in AAA individuals younger than 70 years, whereas no such age-dependent effect was seen in the general population (Figure 1B). The age-related pattern was confirmed in the log-minus-log-plot used to evaluate the proportional hazard assumption in the Cox PH regression model (Supplemental material, Figure S1). The age-dependent pattern in the AAA group remained in the analyses adjusted for potential confounders, as illustrated in predicted survival plots based on the Cox-PH regression models, stratified on HADS-D and AAA to allow for deviation from the PH assumption (Figure 2). HR

For peer review only - http://bmjopen.bmj.com/site/about/guidelines.xhtml

values are not calculated for stratification variables in the Cox PH model. A low number of deaths, as well as a low number of individuals at risk in the subgroup with both AAA and depressive symptoms below the age of 70 years (Figure 1B), precluded an age-specific analysis.

For peer review only

Table 1. Demographic and clinical characteristics at start of follow-up, total and by AAA

Characteristics	Total population n (%)	AAA Yes n (%)	AAA No n (%)	P-value
**Total**	36616 (100)	583 (100)	36033 (100)	
**Sex**				<0.001
Men	17883 (48.8)	432 (74.1)	17451 (48.4)	
Women	18733 (51.2)	151 (25.9)	18582 (51.6)	
**Age at measured HADS-D**				<0.001
<60	20843 (56.9)			
60-69	9550 (26.1)			
70-79	5254 (14.3)			
≥80	969 (2.4)			
**HADS-D**				<0.001
<8	31784 (86.3)	477 (81.8)	31307 (86.9)	
≥8	4832 (13.2)	106 (18.2)	4726 (13.1)	
**Smoking**				<0.001
Never	12890 (35.9)	53 (9.2)	12837 (36.4)	
Past	12738 (35.5)	192 (33.5)	12546 (35.5)	
Current	10260 (28.6)	12546 (35.5)	9931 (28.1)	
**BMI**				0.26
<25	11198 (30.8)	177 (30.7)	11021 (30.8)	
25-29	17349 (47.8)	280 (48.5)	17069 (47.7)	
30-35	6107 (16.8)	103 (17.8)	6004 (16.8)	
≥35	1679 (4.6)	17 (3.0)	1662 (4.7)	
**CHD**	3347 (9.2)	423 (27.4)	160 (8.9)	<0.001
**Hypertension**	21124 (57.7)	423 (72.5)	20701 (57.5)	<0.001
**Diabetes**	1746 (4.8)	23 (4.0)	1723 (4.8)	0.09
**Civil status**				0.35
Cohabiting	26650 (72.9)	443 (76.0)	26207 (72.8)	
Living alone	9913 (27.1)	140 (24.0)	9773 (27.2)	

AAA = Abdominal Aortic Aneurysm, HADS = Hospital Anxiety and Depression Scale, D = depression subscale, BMI = Body Mass Index,
Missing data among those with valid HADS-D (n = 36616): Smoking, n = 728 (2.6%) BMI, n = 283 (0.8%), coronary heart disease, n = 23 (0.06%), systolic and diastolic blood pressure, n = 154 (0.4%), hypertension, n = 9 (0.02%), diabetes, n = 84 (0.2%), civil status, n = 53 (0.14%). Missing data on HADS-D, n = 5259 (12.6%).

For peer review only

Table 2 Hazard ratios (HR) of death for depressive symptoms (HADS-D ≥ 8 vs < 8) and AAA (yes vs. no), based on Cox PH regression model*

	No. of deahts	Unadjusted HR (95% CI)	Adjusted HR (95% CI)**
**AAA**			
No	9078	1.00	1.00
Yes	350	3.39 (3.01-3.78)	2.66 (2.39-2.97)
**HADS-D**			
Individuals with AAA			
<8	283	1.00	1.00
≥8	67	1.28 (0.98-1.69)	1.15 (0.88-1.51)
Individuals without AAA			
<8	7430	1.00	1.00
≥8	1648	1.28 (1.21-1.36)	1.23 (1.17-1.30)
**P-value, interaction**		0.90	0.62

Depressive symptoms were defined as a HADS-D score ≥8.
AAA = Abdominal Aortic Aneurysm, HR = Hazard ratio, CI = Confidence Interval, HADS= Hospital Anxiety and Depression Scale.
*HR from multiple Cox PH regression model with attained age as time scale, adjusted for HADS-D (<8 vs. ≥8), AAA, sex, coronary heart disease, diabetes mellitus, body mass index - category, smoking, hypertension, civil status.
**Complete case analyses: Number of individuals, n= 34464.

For peer review only - http://bmjopen.bmj.com/site/about/guidelines.xhtml

DISCUSSION

In this large, population-based prospective study, the overall risk of death was more than twice as high in individuals with AAA compared to a general population at the same age and same risk profile. There was no adverse effect of depressive symptoms on all-cause mortality in individuals diagnosed with AAA, whereas a negative prognostic effect was observed in the general population. At ages <70 years, however, there was a tendency towards a more pronounced adverse effect of depressive symptoms in individuals with AAA. This finding was uncertain due to the low number of deaths before the age of 70 years.

The finding of a substantially higher mortality in individuals with vs. without AAA is consistent with a recent study reporting markedly lower overall long-term survival in patients undergoing successful AAA repair as compared to age-and sex-adjusted mortality rates in the general population.[27] An unfavorable co-morbidity profile commonly seen in AAA patients, including other cardiovascular diseases, as well as smoking, is probably a contributing factor to the high crude mortality rates in individuals with AAA.[28] In our study, however, the relative risk estimate remained high also after adjusting for factors associated with both AAA and increased mortality, such as sex, coronary heart disease, diabetes and smoking. We are not aware of any previous studies that have compared mortality in individuals with AAA with a general population of the same age and also same level of other risk factors. Most previous studies have almost exclusively been restricted to patients that have undergone surgery for AAA or patients deemed unfit for surgery.[27, 29-31] The present study included both surgically untreated and treated AAA patients, but we did not distinguish between these subgroups in the analyses due to lack of information on this issue.

For peer review only - http://bmjopen.bmj.com/site/about/guidelines.xhtml

To our knowledge, no previous studies have examined whether depressive symptoms on mortality differ in patients with AAA compared to a general population. The results from the present study did not confirm our initial hypothesis that a negative prognostic impact of depressive symptoms may be stronger in individuals with AAA compared to a general population. On the contrary, there was no association between depressive symptoms and mortality in the AAA group. The lack of association could relate to the low number of individuals with both AAA and depressive symptoms. However, we did observe a slightly more pronounced adverse effect of depressive symptoms in individuals diagnosed with AAA before the age of 70 years, whereas no such age-dependent effect was seen in the general population. This pattern may relate to the fact that some risk factors for AAA, such as a high number of pack years (smoking) and unfavorable heredity patterns have been reported to be more common in young compared to older AAA patients.[32] We did adjust for smoking habits, whereas data on heritability was not available. Due to the low number of individuals in these age groups, these results must be interpreted with caution and confirmed in other studies.

The biological mechanisms behind the increased mortality rates in individuals with AAA are yet to be clarified. Inflammation seems to be an important part of both the AAA pathogenesis and growth,[33, 34] which may relate to impact from noxious stimuli such as cigarette smoke, low density lipoprotein cholesterol and hypertension.[35] With increasing age, competent repair cells are not always available, artery repair fails and the inflammatory state increases.[1] Previous studies have shown that depression is associated with low-grade chronic inflammation.[1, 15, 36] Moreover, a negative impact from inflammatory processes on mortality in patients with CVDs and concurrent depression has been suggested.[37] Our

For peer review only - http://bmjopen.bmj.com/site/about/guidelines.xhtml

finding indicating that depressive symptoms might worsen the prognosis in younger, but not in older individuals diagnosed with AAA might be somewhat contradictory. On the other hand, it is possible that individuals who develop AAA at a younger age have faster aneurysm growth that is more susceptible to a growth-enhancing effect from depression, potentially mediated by inflammation.

Strengths and limitations

A major strength of our study is that the results are based on data from a large, population-based, prospective study. The HUNT population is a validated and well-defined homogenous cohort that represents the general population in Norway. Data on risk factors were assessed at the individual level for all study participants, enabling tight adjustment for age (1- year intervals, time scale in the survival analyses), sex and other potential confounders. Repeated measurements of the exposure factors made it possible to update the risk set by use of time-dependent covariates. The use of time-varying covariates thus reduced potential misclassification bias in the risk estimates. However, not all the individuals included in our study had participated in HUNT more than once, which is a limitation. With age as the time scale in the survival analyses, a detailed adjustment for age was made in the estimation of HR values. The amount of missing data for the covariates was low. All AAA cases were verified based on a certified hospital diagnosis and dates of death were obtained from national registers with compulsory recordings. It is, however, likely that there was some underdiagnosis of AAA cases. The HUNT study does not include ultrasound examinations of the abdominal aorta. The observed 1.6% point prevalence of AAA in our study is, however, in line with the expected rate at the time.[38] Unless a potential underdiagnosis of AAA differ by value of HADS-D, the risk estimates will not be biased. Some limitations must be considered.

For peer review only - http://bmjopen.bmj.com/site/about/guidelines.xhtml

Most data on covariates were self-reported, including symptoms of depression, cardiovascular comorbidities and smoking, potentially leading to information bias. Symptoms of depression were assessed through self-reported questionnaires and not by a psychiatric diagnostic interview. We had no information about antidepressant use. The low number of individuals with severe depressive symptoms in our study population could be a result from recruitment bias. Individuals with severe depression might not adhere to screening programs or attend population-based studies to the same extent as non-depressed individuals. However, such recruitment bias would likely bias our results towards the null hypothesis.

CONCLUSION

In this population-based prospective study there was no significant prognostic impact of depressive symptoms on mortality in individuals with AAA, whereas an increased risk was seen in the general population. Our results revealed a hitherto unreported higher all-cause mortality in individuals with AAA compared to a general population of the same age and same risk profile. More studies with larger AAA cohorts are needed to explore the relation between depression, AAA and mortality, and a potential heterogeneity across age groups.

Acknowledgements

The Trøndelag Health Study (The HUNT Study) is a collaboration between HUNT Research Centre, (Faculty of Medicine and Health Sciences, NTNU, Norwegian University of Science and Technology), Trøndelag County Council, Central Norway Regional Health Authority, and the Norwegian Institute of Public Health.

For peer review only

Contributorship

LN contributed to study conception and design, data and statistical analysis, interpretation of study findings and drafting and critical review of the manuscript. RH contributed to study conception and design, interpretation of study findings and drafting and critical review of the manuscript.

GA contributed to study conception, and statistical methods and analysis, interpretation of study findings and critical review of the manuscript. EM contributed to study conception and design and critical review of the manuscript. MS contributed to study conception and design, interpretation of study findings and drafting and critical review of the manuscript.

Data availability statement

No data are available

The Trøndelag Health Study (HUNT) has invited persons aged 13 - 100 years to four surveys between 1984 and 2019. Comprehensive data from more than 140.000 persons having participated at least once and biological material from 78.000 persons are collected. The data are stored in HUNT databank and biological material in HUNT biobank. HUNT Research Centre has permission from the Norwegian Data Inspectorate to store and handle these data. The key identification in the data base is the personal identification number given to all Norwegians at birth or immigration, whilst de-identified data are sent to researchers upon approval of a research protocol by the Regional Ethical Committee and HUNT Research Centre. To protect participants' privacy, HUNT Research Centre aims to limit storage of data outside HUNT databank, and cannot deposit data in open repositories. HUNT databank has precise information on all data exported to different projects and are able to reproduce

For peer review only - http://bmjopen.bmj.com/site/about/guidelines.xhtml

these on request. There are no restrictions regarding data export given approval of applications to HUNT Research Centre. For more information see: https://eur01.safelinks.protection.outlook.com/?url=http%3A%2F%2Fwww.ntnu.edu%2Fhunt%2Fdata&data=04%7C01%7Cmalin.stenman%40ki.se%7C1bc54c35ed5f4ce79e0508d8a7616b52%7Cbff7eef1cf4b4f32be3da1dda043c05d%7C0%7C0%7C637443384157146991%7CUnknown%7CTWFpbGZsb3d8eyJWIjoiMC4wLjAwMDAiLCJQIjoiV2luMzIiLCJBTiI6Ik1haWwiLCJXVCI6Mn0%3D%7C1000&sdata=GwGqQpTG4u%2FoslrgO43q64XHIpvyeEMTSCo%2ByrTn7i4%3D&reserved=0

Competing interests

None declared

Funding

This research has been supported by the Swedish Heart-Lung Foundation (Hultgren) grant number: 20190553 and 20180506.

Patient consent for publication

Not required

Ethics approval

The study was approved by the HUNT Study board of directors and the Regional Ethics Committee (2014/175/REK12 Midt-Norge).

Disclosures

The authors of this manuscript have no potential conflicts of interest.

For peer review only

REFERENCES

1. Sakalihasan N, Michel JB, Katsargyris A, et al. Abdominal aortic aneurysms. *Nat Rev Dis Primers* 2018;4:34.
2. Hare DL, Toukhsati SR, Johansson P, et al. Depression and cardiovascular disease: a clinical review. *Eur Heart J* 2014;35:1365-72.
3. Bath MF, Sidloff D, Saratzis A, et al. Impact of abdominal aortic aneurysm screening on quality of life. *Br J Surg* 2018;105:203-08.
4. Wanhainen A, Hultgren R, Linne A, et al. Outcome of the Swedish Nationwide Abdominal Aortic Aneurysm Screening Program. *Circulation* 2016;134:1141-48.
5. Norman PE, Jamrozik K, Lawrence-Brown MM, et al. Population based randomised controlled trial on impact of screening on mortality from abdominal aortic aneurysm. *Bmj* 2004;329:1259.
6. Ashton HA, Buxton MJ, Day NE, et al. The Multicentre Aneurysm Screening Study (MASS) into the effect of abdominal aortic aneurysm screening on mortality in men: a randomised controlled trial. *Lancet* 2002;360:1531-9.
7. Lindholt JS, Juul S, Fasting H, et al. Screening for abdominal aortic aneurysms: single centre randomised controlled trial. *Bmj* 2005;330:750.
8. Ali MU, Fitzpatrick-Lewis D, Miller J, et al. Screening for abdominal aortic aneurysm in asymptomatic adults. *J Vasc Surg* 2016;64:1855-68.
9. Timmers TK, van Herwaarden JA, de Borst GJ, et al. Long-term survival and quality of life after open abdominal aortic aneurysm repair. *World J Surg* 2013;37:2957-64.
10. de Bruin JL, Baas AF, Heymans MW, et al. Statin therapy is associated with improved survival after endovascular and open aneurysm repair. *J Vasc Surg* 2014;59:39-44.e1.
11. Bath MF, Gokani VJ, Sidloff DA, et al. Systematic review of cardiovascular disease and cardiovascular death in patients with a small abdominal aortic aneurysm. *Br J Surg* 2015;102:866-72.
12. Mykletun A, Bjerkeset O, Overland S, et al. Levels of anxiety and depression as predictors of mortality: the HUNT study. *Br J Psychiatry* 2009;195:118-25.
13. Harshfield EL, Pennells L, Schwartz JE, et al. Association Between Depressive Symptoms and Incident Cardiovascular Diseases. *Jama* 2020;324:2396-405.
14. Stenman M, Holzmann MJ, Sartipy U. Relation of major depression to survival after coronary artery bypass grafting. *Am J Cardiol* 2014;114:698-703.
15. Nemeroff CB, Goldschmidt-Clermont PJ. Heartache and heartbreak--the link between depression and cardiovascular disease. *Nat Rev Cardiol* 2012;9:526-39.
16. Daskalopoulou M, George J, Walters K, et al. Depression as a Risk Factor for the Initial Presentation of Twelve Cardiac, Cerebrovascular, and Peripheral Arterial Diseases: Data Linkage Study of 1.9 Million Women and Men. *PLoS One* 2016;11:e0153838.
17. Nyronning LA, Stenman M, Hultgren R, et al. Symptoms of Depression and Risk of Abdominal Aortic Aneurysm: A HUNT Study. *J Am Heart Assoc* 2019;8:e012535.
18. Pan A, Sun Q, Okereke OI, et al. Depression and risk of stroke morbidity and mortality: a meta-analysis and systematic review. *Jama* 2011;306:1241-9.
19. Sakalihasan N, Van Damme H, Gomez P, et al. Positron emission tomography (PET) evaluation of abdominal aortic aneurysm (AAA). *Eur J Vasc Endovasc Surg* 2002;23:431-6.

For peer review only - http://bmjopen.bmj.com/site/about/guidelines.xhtml

20. Dale MA, Ruhlman MK, Baxter BT. Inflammatory cell phenotypes in AAAs: their role and potential as targets for therapy. *Arterioscler Thromb Vasc Biol* 2015;35:1746-55.
21. Almeida OP, Ford AH, Hankey GJ, et al. Depression, antidepressants and the risk of cardiovascular events and death in older men. *Maturitas* 2019;128:4-9.
22. Cuijpers P, Vogelzangs N, Twisk J, et al. Comprehensive meta-analysis of excess mortality in depression in the general community versus patients with specific illnesses. *Am J Psychiatry* 2014;171:453-62.
23. Letnes JM, Torske MO, Hilt B, et al. Symptoms of depression and all-cause mortality in farmers, a cohort study: the HUNT study, Norway. *BMJ Open* 2016;6:e010783.
24. Krokstad S, Langhammer A, Hveem K, et al. Cohort Profile: the HUNT Study, Norway. *Int J Epidemiol* 2013;42:968-77.
25. Bjelland I, Dahl AA, Haug TT, et al. The validity of the Hospital Anxiety and Depression Scale. An updated literature review. *J Psychosom Res* 2002;52:69-77.
26. Myrtveit SM, Ariansen AM, Wilhelmsen I, et al. A population based validation study of self-reported pensions and benefits: the Nord-Trondelag health study (HUNT). *BMC Res Notes* 2013;6:27.
27. Bulder RMA, Talvitie M, Bastiaannet E, et al. Long-term Prognosis After Elective AAA Repair is Poor in Women and Men: The Challenges Remain. *Ann Surg* 2020.
28. Toghill BJ, Saratzis A, Bown MJ. Abdominal aortic aneurysm-an independent disease to atherosclerosis? *Cardiovasc Pathol* 2017;27:71-75.
29. Whittaker JD, Meecham L, Summerour V, et al. Outcome after Turndown for Elective Abdominal Aortic Aneurysm Surgery. *Eur J Vasc Endovasc Surg* 2017;54:579-86.
30. Rueda-Ochoa OL, van Bakel P, Hoeks SE, et al. Survival After Uncomplicated EVAR in Octogenarians is Similar to the General Population of Octogenarians Without an Abdominal Aortic Aneurysm. *Eur J Vasc Endovasc Surg* 2020;59:740-47.
31. Norman PE, Semmens JB, Lawrence-Brown MM, et al. Long term relative survival after surgery for abdominal aortic aneurysm in western Australia: population based study. *Bmj* 1998;317:852-6.
32. Howard DP, Banerjee A, Fairhead JF, et al. Age-specific incidence, risk factors and outcome of acute abdominal aortic aneurysms in a defined population. *Br J Surg* 2015;102:907-15.
33. Kuivaniemi H, Ryer EJ, Elmore JR, et al. Understanding the pathogenesis of abdominal aortic aneurysms. *Expert Rev Cardiovasc Ther* 2015;13:975-87.
34. Eagleton MJ. Inflammation in abdominal aortic aneurysms: cellular infiltrate and cytokine profiles. *Vascular* 2012;20:278-83.
35. Rocha VZ, Libby P. Obesity, inflammation, and atherosclerosis. *Nat Rev Cardiol* 2009;6:399-409.
36. Raedler TJ. Inflammatory mechanisms in major depressive disorder. *Curr Opin Psychiatry* 2011;24:519-25.
37. Celano CM, Huffman JC. Depression and cardiac disease: a review. *Cardiol Rev* 2011;19:130-42.
38. Ulug P, Powell JT, Sweeting MJ, et al. Meta-analysis of the current prevalence of screen-detected abdominal aortic aneurysm in women. *Br J Surg* 2016;103:1097-104.

For peer review only

Figure legends

Figure 1.
Kaplan-Meier estimates of proportion of deaths and numbers of individuals at risk at different ages during follow-up in AAA patients and in the general population and Kaplan-Meier estimates of proportion of deaths in combined categories of AAA (no, yes) and HADS-D (<8 versus ≥8)

Figure 2. Expected proportion of deaths in combined categories of AAA (no, yes) and HADS-D (<8 versus ≥8), based on a stratified Cox PH regression model. A. Unadjusted. B. Adjusted for age (time-scale), sex, BMI category, smoking, CHD, diabetes mellitus, civil status and hypertension.

For peer review only

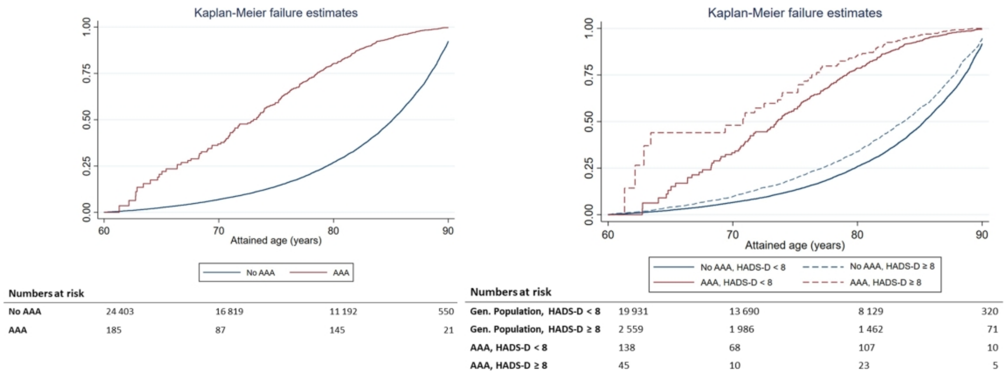

Figure 1.
Kaplan-Meier estimates of proportion of deaths and numbers of individuals at risk at different ages during follow-up in AAA patients and in the general population and Kaplan-Meier estimates of proportion of deaths in combined categories of AAA (no, yes) and HADS-D (<8 versus ≥8)

335x121mm (300 x 300 DPI)

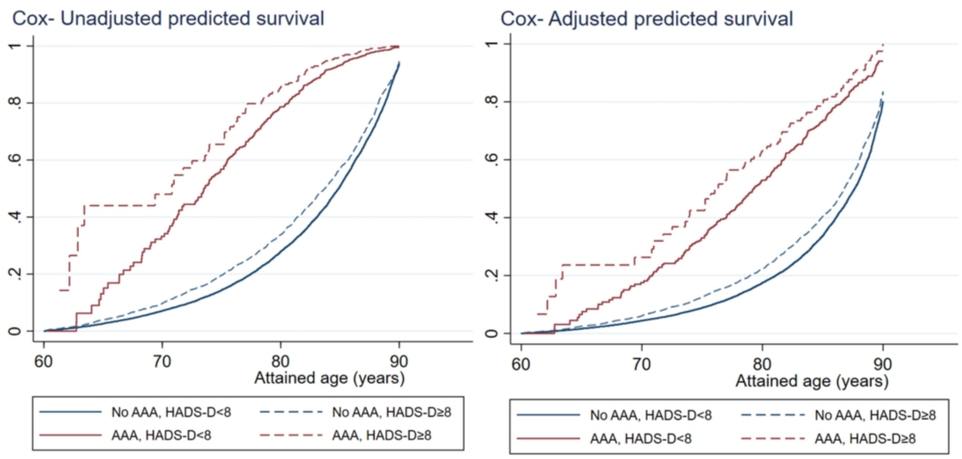

Figure 2. Expected proportion of deaths in combined categories of AAA (no, yes) and HADS-D (<8 versus ≥8), based on a stratified Cox PH regression model. A. Unadjusted. B. Adjusted for age (time-scale), sex, BMI category, smoking, CHD, diabetes mellitus, civil status and hypertension.

338x190mm (300 x 300 DPI)

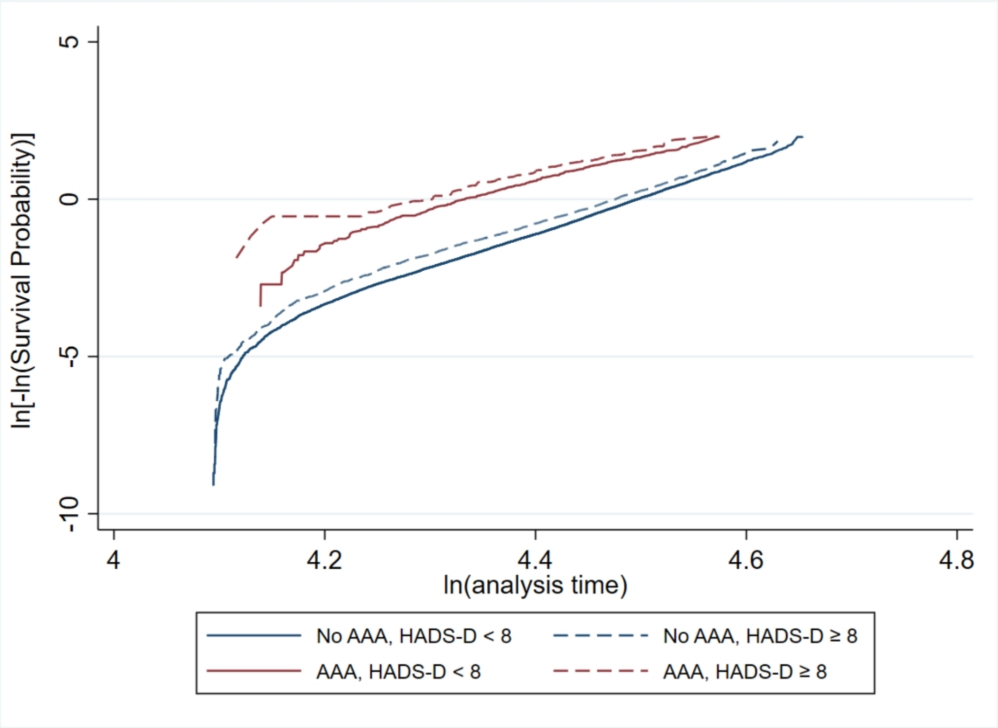

Figure S1. "Log - log plot" to test the proportional hazards assumption. If the plotted lines are reasonably parallel, the proportional hazards assumption has not been violated.

415x302mm (300 x 300 DPI)

STROBE 2007 (v4) Statement—Checklist of items that should be included in reports of *cohort studies*

Section/Topic	Item #	Recommendation	Reported on page #
**Title and abstract**	1	(*a*) Indicate the study's design with a commonly used term in the title or the abstract	1
		(*b*) Provide in the abstract an informative and balanced summary of what was done and what was found	2
**Introduction**			
Background/rationale	2	Explain the scientific background and rationale for the investigation being reported	4-5
Objectives	3	State specific objectives, including any prespecified hypotheses	5
**Methods**			
Study design	4	Present key elements of study design early in the paper	5
Setting	5	Describe the setting, locations, and relevant dates, including periods of recruitment, exposure, follow-up, and data collection	6-8
Participants	6	(*a*) Give the eligibility criteria, and the sources and methods of selection of participants. Describe methods of follow-up	6
		(*b*) For matched studies, give matching criteria and number of exposed and unexposed	6
Variables	7	Clearly define all outcomes, exposures, predictors, potential confounders, and effect modifiers. Give diagnostic criteria, if applicable	6-9
Data sources/ measurement	8*	For each variable of interest, give sources of data and details of methods of assessment (measurement). Describe comparability of assessment methods if there is more than one group	7-8
Bias	9	Describe any efforts to address potential sources of bias	9
Study size	10	Explain how the study size was arrived at	6
Quantitative variables	11	Explain how quantitative variables were handled in the analyses. If applicable, describe which groupings were chosen and why	7-9
Statistical methods	12	(*a*) Describe all statistical methods, including those used to control for confounding	8-9
		(*b*) Describe any methods used to examine subgroups and interactions	9
		(*c*) Explain how missing data were addressed	9
		(*d*) If applicable, explain how loss to follow-up was addressed	
		(*e*) Describe any sensitivity analyses	9
**Results**			

For peer review only

Participants	13*	(a) Report numbers of individuals at each stage of study—eg numbers potentially eligible, examined for eligibility, confirmed eligible, included in the study, completing follow-up, and analysed	6,7,11
		(b) Give reasons for non-participation at each stage	
		(c) Consider use of a flow diagram	
Descriptive data	14*	(a) Give characteristics of study participants (eg demographic, clinical, social) and information on exposures and potential confounders	11
		(b) Indicate number of participants with missing data for each variable of interest	14
		(c) Summarise follow-up time (eg, average and total amount)	7
Outcome data	15*	Report numbers of outcome events or summary measures over time	11-12
Main results	16	(*a*) Give unadjusted estimates and, if applicable, confounder-adjusted estimates and their precision (eg, 95% confidence interval). Make clear which confounders were adjusted for and why they were included	11-13
		(*b*) Report category boundaries when continuous variables were categorized	8, 14
		(*c*) If relevant, consider translating estimates of relative risk into absolute risk for a meaningful time period	
Other analyses	17	Report other analyses done—eg analyses of subgroups and interactions, and sensitivity analyses	11-13
**Discussion**			
Key results	18	Summarise key results with reference to study objectives	16
**Limitations**			16-19
Interpretation	20	Give a cautious overall interpretation of results considering objectives, limitations, multiplicity of analyses, results from similar studies, and other relevant evidence	19
Generalisability	21	Discuss the generalisability (external validity) of the study results	19
**Other information**			
Funding	22	Give the source of funding and the role of the funders for the present study and, if applicable, for the original study on which the present article is based	21

*Give information separately for cases and controls in case-control studies and, if applicable, for exposed and unexposed groups in cohort and cross-sectional studies.

Note: An Explanation and Elaboration article discusses each checklist item and gives methodological background and published examples of transparent reporting. The STROBE checklist is best used in conjunction with this article (freely available on the Web sites of PLoS Medicine at http://www.plosmedicine.org/, Annals of Internal Medicine at http://www.annals.org/, and Epidemiology at http://www.epidem.com/). Information on the STROBE Initiative is available at www.strobe-statement.org.

For peer review only

BMJ Open

The prognostic impact of depressive symptoms on all-cause mortality in individuals with abdominal aortic aneurysm and in the general population. A population-based prospective HUNT study in Norway

Journal:	*BMJ Open*
Manuscript ID	bmjopen-2021-049055.R2
Article Type:	Original research
Date Submitted by the Author:	30-Aug-2021
Complete List of Authors:	Nyrønning, Linn Å.; Department of Surgery, Vascular Surgery; NTNU Department of Circulation and Medical Imaging<br>Hultgren, Rebecka; Karolinska Institute, Deparment of molecular medicine and surgery; Karolinska Universitetssjukhuset, Department of Vascular Surgery<br>Albrektsen, Grethe; Norges teknisk-naturvitenskapelige universitet, Department of public health and general practice<br>Mattsson, Erney; Department of Surgery, Vascular Surgery; NTNU Department of Circulation and Medical Imaging<br>Stenman, Malin ; Karolinska Institutet, Department of Molecular Medicine and Surgery; Karolinska University Hospital, Department of Molecular Medicine and Surgery
**Primary Subject Heading**:	Epidemiology
Secondary Subject Heading:	Epidemiology, Cardiovascular medicine
Keywords:	EPIDEMIOLOGY, Vascular medicine < INTERNAL MEDICINE, Vascular surgery < SURGERY, VASCULAR SURGERY

SCHOLARONE™
Manuscripts

I, the Submitting Author has the right to grant and does grant on behalf of all authors of the Work (as defined in the below author licence), an exclusive licence and/or a non-exclusive licence for contributions from authors who are: i) UK Crown employees; ii) where BMJ has agreed a CC-BY licence shall apply, and/or iii) in accordance with the terms applicable for US Federal Government officers or employees acting as part of their official duties; on a worldwide, perpetual, irrevocable, royalty-free basis to BMJ Publishing Group Ltd ("BMJ") its licensees and where the relevant Journal is co-owned by BMJ to the co-owners of the Journal, to publish the Work in this journal and any other BMJ products and to exploit all rights, as set out in our licence.

The Submitting Author accepts and understands that any supply made under these terms is made by BMJ to the Submitting Author unless you are acting as an employee on behalf of your employer or a postgraduate student of an affiliated institution which is paying any applicable article publishing charge ("APC") for Open Access articles. Where the Submitting Author wishes to make the Work available on an Open Access basis (and intends to pay the relevant APC), the terms of reuse of such Open Access shall be governed by a Creative Commons licence – details of these licences and which Creative Commons licence will apply to this Work are set out in our licence referred to above.

Other than as permitted in any relevant BMJ Author's Self Archiving Policies, I confirm this Work has not been accepted for publication elsewhere, is not being considered for publication elsewhere and does not duplicate material already published. I confirm all authors consent to publication of this Work and authorise the granting of this licence.

For peer review only

The prognostic impact of depressive symptoms on all-cause mortality in individuals with abdominal aortic aneurysm and in the general population. A population-based prospective HUNT study in Norway

Linn Å. Nyrønning[1,2] Rebecka Hultgren[3,4] Grethe Albrektsen[5,6], Erney Mattsson[1,2]
Malin Stenman[3,7]

[1]Department of Surgery, Vascular Surgery, St Olavs Hospital, Trondheim, Norway,
[2]Department of Circulation and Medical Imaging, NTNU, Trondheim, Norway,
[3]Department of Molecular Medicine and Surgery, Karolinska Institutet, Stockholm, Sweden,
[4]Department of Vascular Surgery, Karolinska University Hospital, Stockholm, Sweden,
[5]Department of Public Health and Nursing, NTNU, Trondheim, Norway,
[6]Department of clinical and molecular medicine, NTNU, Trondheim, Norway,
[7]Perioperative Medicine and Intensive Care Function, Karolinska University Hospital, Stockholm, Sweden

Corresponding author:
Malin Stenman
Perioperative Medicine and Intensive Care Function
Karolinska University Hospital / Karolinska Institutet
SE-171 76 Stockholm, Sweden
E-mail: Malin.Stenman@ki.se

Word count: 3549

For peer review only

ABSTRACT

Background Abdominal aortic aneurysm (AAA) is a potentially life-threatening disease but the high mortality rate is linked to high age and comorbidity pattern. Depression is associated with increased mortality in the general population and individuals with cardiovascular diseases, but this is sparsely studied for AAA. The aim was to examine the prognostic impact of depressive symptoms on all-cause mortality in individuals with AAA and compare with findings in a general population of the same age and risk profile.

Methods Population-based prospective study including 36616 participants (52.1% women) from the Trøndelag Health Study (HUNT) in Norway. A total of 9428 individuals died during a median follow-up of 10 years at ages 60-90 years. Depressive symptoms were defined by a Hospital Anxiety and Depression (HADS-D) score ≥ 8. Data on AAA diagnoses and death were obtained from medical records and national registers. Hazard ratios (HR) from Cox proportional hazard regression models are reported.

Results A total of 4832 (13.2%) individuals reported depressive symptoms, whereas 583 (1.6%) AAAs were identified. The adjusted hazard of death was 2.66 times higher in persons with AAA compared to the general population (95%CI 2.39-2.97). Overall, there was no significant adverse effect of depressive symptoms in individuals with AAA (HR=1.15;95% CI 0.88-1.51), whereas an increased risk was seen in the general population (HR=1.23;95% CI 1.17-1.30).

Conclusion The overall risk of death was considerably higher in individuals with AAA compared to a general population of the same age and risk profile. Depressive symptoms did not significantly influence the risk of death in the AAA group.

For peer review only

Strengths and limitations of this study

- The results from this study are based on data from a large and population-based prospective study in Norway.
- The general population served as comparison group when evaluating the prognostic impact of depressive symptoms on all-cause mortality in individuals diagnosed with abdominal aortic aneurysm.
- The risk estimates were adjusted for potential confounders, such as age, sex, coronary heart disease, diabetes, BMI, hypertension and civil status.

- All AAA cases were verified based on a certified hospital diagnosis, but due to the lack of ultrasound measurement in HUNT, the prevalence of AAA in the general population may have been underestimated.
- Data on depressive symptoms and comorbidity patterns were self-reported.

For peer review only

INTRODUCTION

Abdominal aortic aneurysm (AAA) is a localized widening of the abdominal aorta that often is asymptomatic and undiagnosed.[1] The most feared complication of an AAA is rupture, a life-threatening bleeding associated with fatal outcome if untreated.[1] To prevent deaths from a ruptured AAA, an early diagnosis and treatment in due time is essential. AAA is in general a late onset disease, that is associated with an unfavorable co-morbidity pattern and high mortality rates. [2, 3] Screening of men aged 65 years or older seems to reduce the risk of deaths related to AAA by up to 30 to 40 %.[4-7] whereas the effect on all-cause mortality is less clear.[8] Nonetheless, mortality rates in patients that have undergone surgery for AAA have been found to be higher than for patients with other cardiovascular diseases (CVDs). [9, 10] High mortality in patients diagnosed with AAA has been linked to high age and unfavorable comorbidity patters, including death from cardiovascular diseases and other smoking-related diseases. Many individuals diagnosed with AAA live with an asymptomatic aneurysm or undergo elective surgery, and eventually die from other causes.[11] Very few previous studies have compared the overall mortality in individuals diagnosed with AAA to a general population of the same age and with the same risk profile.

Depression is associated with increased mortality in the general population.[12] Moreover, depression is common in patients with cardiovascular disease [13] and is independently associated with increased cardiovascular morbidity and mortality.[2, 14, 15] In two recent large prospective, population-based studies, depression was associated with increased risk of AAA.[16, 17] AAA and depression have both been associated with low-grade inflammation.[12, 18] Hence, inflammation could be a common risk factor that may explain the observed association between AAA and depression. Alternatively, the inflammation

For peer review only

process evolves on the pathway between depression and the risk of AAA. Previous studies have suggested that inflammation may increase the risk of rupture by triggering a more rapid growth of the aneurysm.[19, 20] If depression, as a proxy for low grade inflammation, influences both the risk and progression of AAA development, an adverse prognostic impact of depression may be particularly pronounced in individuals with AAA. An adverse effect of depression on mortality rates in patients with other cardiovascular diseases has been established [12, 15, 18, 21-23], but few previous studies have examined the prognostic impact of depression in individuals with AAA. Moreover, we are not aware of any previous studies that have compared with findings in a general population.

The main aim of the present study was to examine whether the prognostic impact of depressive symptoms on all-cause mortality in individuals diagnosed with AAA differed from the established adverse effect in the general population. Moreover, we investigate whether there was an overall difference in all-cause mortality between individuals diagnosed with AAA and the general population of the same age and with the same risk profile.

METHODS

This is a prospective population-based cohort study, based on data obtained from the Norwegian Trøndelag Health Study (HUNT). The study has been approved by the HUNT Study board of directors and the Regional Ethics Committee (2014/175/REK12 Midt-Norge), and was conducted in accordance with the Helsinki Declaration. The study was performed following the STROBE checklist for cohort studies (supplemental material).

For peer review only - http://bmjopen.bmj.com/site/about/guidelines.xhtml

The HUNT Study

HUNT is a large and ongoing multiphase population-based health study of people aged 20 years or older conducted in the county of Nord-Trøndelag in central Norway.[24] The databank contains extensive information about a wide variety of demographics and health-related topics. Data on depressive symptom was introduced in the second HUNT survey (HUNT2).

Study population

The present study population includes 36616 individuals (52.1 % women) who participated in HUNT2 and/or HUNT3 and had available data in HUNT on depressive symptoms. Median age at the first participation in HUNT was 58.6 years (range 40.6-89.8 years). The unique personal identification number was used to link data on AAA diagnoses (ICD-9/10) and death with data from the HUNT study.

Patient and Public Involvement

This study has no direct contact with patients or the public. The study utilize de-identified data from the HUNT study.

Primary outcome and follow-up period

The primary outcome in this study was all-cause mortality. The Norwegian Cause of Death Registry was used to acquire information on dates of death. Each individual was considered to be at risk of death from the age of 60 years, or from the date of first HUNT participation at a higher age, until date of death or, reaching the age of 90 years, or closing date of the study

For peer review only - http://bmjopen.bmj.com/site/about/guidelines.xhtml

(December 31, 2014). The lower and upper age limits were defined due to few deaths before the age of 60 years and few individuals with AAA at risk above the age of 90 years. Median age at start of follow-up was 60 years (range 60.0- 89.8 years) and median follow-up time was 10 years (1.1 days to 20 years). At the end of the study period, the median age was 73 years (range 60-90 years). Among the 36616 individuals included in the study (51.2 % women), 9428 (44.0 % women) died during follow-up.

Ascertainment of AAA

Information on the diagnosis of AAA in the period 1995 through 2014 was obtained from local hospital registers (in-hospital and outpatient). An indicator variable for whether or not a person had been diagnosed with AAA was defined according to codes 441.3-4 and I71.3-4 in the ninth and tenth editions of the International Classification of Diseases (ICD 9/10 codes). All AAA diagnoses were manually verified using patient charts.

Assessment of depressive symptoms

The Hospital Anxiety and Depression Scale (HADS) was used to assess the degree of depressive symptoms. HADS is a self-report questionnaire developed to evaluate patients in somatic care and aims to measure levels of anxiety and depression symptoms during the preceding week. The questionnaire consists of two separate subscales ranging from 0-21: the HADS Anxiety (HADS-A) and HADS Depression (HADS-D). Only the HADS-D score was used in the present study. A HADS-D score ≥ 8 was defined as having depressive symptoms. A cut-off at ≥ 8 (HADS-D ≥8) gives a specificity of 0.79 and a sensitivity of 0.83 compared with a clinical evaluation of depression.[25, 26]

For peer review only - http://bmjopen.bmj.com/site/about/guidelines.xhtml

Demographic and clinical factors

Information on potential confounders was assessed through responses on self-reported questionnaires and clinical examinations. Sex and civil status (categorized as living alone or with other) were assessed through questionnaires. Hypertension (yes, no) was defined as systolic blood pressure > 140 mmHg, diastolic blood pressure > 90 mmHg (average of second and third measurements) or use of antihypertensive medication (self-reported). Body mass index (BMI) was calculated as weight/height^2 (kg/m^2), and divided into four categories (<25, 25-30, 30-35, ≥35 kg/m^2). Coronary heart disease (CHD, yes, no) was defined as a composite variable of self-reported history of myocardial infarction or angina pectoris. Diabetes (yes, no), smoking status (never, past, current) were defined according to questionnaire responses.

Statistical analysis

Descriptive statistics at start of follow-up are presented as mean (standard deviation, SD), median (interquartile range), and/or as frequencies (%), according to nature of the data. The Kaplan-Meier approach was applied to calculate the expected proportion of non-survivors (proportion of deaths) at different ages for AAA (yes, no), and for combined categories of the HADS-D score (<8, ≥8) and the indicator variable for AAA (yes, no). The log-rank test was applied to compare the overall survival between the groups. In addition, the Nelson-Aalen estimate of the cumulative hazard function is presented. The shape of the curves in these plots is informative with respect to changes in hazard with increasing age in each exposure group. Unadjusted and adjusted hazard ratios (HR) for death with 95 % confidence intervals (CI) were calculated as estimate of relative risk in a Cox proportional hazard (PH) regression model. A multiplicative interaction term between HADS-D and AAA was added to evaluate

For peer review only - http://bmjopen.bmj.com/site/about/guidelines.xhtml

whether the association between depressive symptoms and all-cause mortality differed for persons with and without AAA. HUNT participants without a diagnosis of AAA were considered representative for the general population. The analyses were adjusted for age (time-scale), sex, BMI category, smoking, CHD, diabetes mellitus, hypertension and civil status. In the analyses of the overall association between AAA and mortality, we also adjusted for depressive symptoms. A backward stepwise procedure was applied to evaluate the individual contributions from single factors and to search for signs of potential overfitting and/or collinearity problems. The proportional hazard (PH) assumption for each covariate in the Cox PH regression model was visually evaluated in log-minus-log plots. Supplementary analyses stratified for adjustment factors that showed signs of deviation from the PH assumption were performed. Predicted survival plots based on the Cox PH regression model are also presented. Individuals with missing values on HADS-D (12.6 %) or other covariates were excluded from the analyses (complete case analysis, n= 34 464).

Time-varying exposure factors

Attained age was defined as time scale in the survival analyses. Except for sex, all prognostic factors were treated as time-varying covariates in the Cox PH regression model, allowing a shift in exposure categories with increasing age for individuals who participated in two HUNT surveys. The covariate values at start of follow-up were defined according to values obtained at the HUNT participation closest in time before the age of 60 years or at the first HUNT participation at an older age. In the Kaplan-Meier survival analyses, the 'at risk' category was defined according to the most recent registered value of the exposure factor during follow-up. The indicator variable for AAA was 0 (not present) until date (age) of diagnosis (recoded to 1, i.e. present). The diagnosis of AAA could occur either before, after or between two HUNT

For peer review only - http://bmjopen.bmj.com/site/about/guidelines.xhtml

participations, depending on age at diagnosis and age at participation in a HUNT survey. A total of 27 individuals (2 women) had been diagnosed with AAA at start of follow-up from the age of 60 years, whereas an additional 556 individuals (149 women) were diagnosed during follow-up.

All statistical analyses were performed using Stata (Version IC.16.1 Stata Corp, College Station, TX). A p value <0.05 was considered significant (2-sided tests).

For peer review only

RESULTS

The distribution of the demographic and clinical characteristics of the study population at start of follow-up is presented in Table 1. A total of 583 (1.6 %) individuals (432 men, 151 women) had a diagnosed AAA, and 4 832 (13.2%) individuals reported depressive symptoms (HADS-D ≥ 8). The proportion with a HADS-D score ≥ 8 was higher in individuals with than without a diagnosed AAA (Table 1). Compared with persons without AAA (considered representative for the general population), individuals with AAA were more often men (74.1 vs. 48.4 %), current smokers (57.5 vs. 35.5 %) and reported more coronary heart disease (27.4 vs. 8.9 %) and hypertension (72.5 vs. 57.5 %). Moreover, a larger proportion in the AAA group was obese (4.7 vs 3.0 %), but no significant association with BMI category was found.

All-cause mortality in individuals with AAA and in the general population

A Kaplan Meier failure plot, with estimated proportion of deaths at different ages, for individuals with and without a diagnosed AAA, and Nelson-Allen estimates of the cumulative hazard function, are shown in Figure 1. Individuals with AAA died at a younger age compared to the general population (median age at death 73.0 vs. 82.5 years, (log rank test, p-value < 0.001). In the Cox PH regression model without adjustment for potential confounders, the hazard of death in individuals with AAA was more than three times the hazard of death in the general population (Table 2, HR 3.39, 95 % CI 3.01-3.78). The difference was less pronounced in the adjusted analysis but the hazard remained more than twice as high in the AAA group (Table 2, HR 2.66, 95 % CI 2.39-2.97).

For peer review only - http://bmjopen.bmj.com/site/about/guidelines.xhtml

Prognostic impact of depression in individuals with AAA and in the general population

At all ages, the estimated proportions of deaths were higher among individuals with than without depressive symptoms (HADS-D ≥8 vs. >8), both in individuals diagnosed with AAA and in the general population (Figure 2). Median age at death in individuals with and without depressive symptoms in the AAA group was 70.8 and 73.5 years, whereas the corresponding numbers in the general population were 83.8 and 85.4 years.

In the Cox PH regression analyses without adjustment for potential confounders, the adverse effect of depressive symptoms in individuals with AAA was rather similar to the association seen in the general population (Table 2, HR for HADS-D ≥8 vs. < 8 of 1.28 in both groups). In the analyses adjusted for sex, smoking, coronary heart disease (CHD), diabetes, BMI, hypertension, and civil status (Table 2) there was no significant prognostic impact if depressive symptoms in individuals with AAA (HR 1.15, 95%CI 0.88-1.51), whereas a weak adverse effect remained in the general population (HR 1.23, 95%CI 1.17-1.30). The difference in the prognostic impact of depressive symptoms between the two groups did not reach statistical significance (p, test for interaction = 0.62). The factors contributing most to the reduced strength of the association with depressive symptoms were CHD, BMI and smoking. There was no single factor that explained the flattening of the risk estimate or led to very imprecise estimates.

HR values (adjusted) for combined categories of HADS-D and AAA, using a common reference group, to allow for a direct comparison of relative risk across all subgroups, are shown in Figure 3. The adverse, though weak association with depression appeared to be rather similar

For peer review only

in individuals with and without AAA in this analysis. The prognostic impact of AAA was considerably more pronounced than the prognostic impact of depressive symptoms

Age-dependent effects

The unadjusted Kaplan Meier failure plot, as well as the Nelson-Aalen cumulative hazard plot (Figure 2), indicated a more pronounced negative prognostic effect from depressive symptoms in AAA individuals younger than 70 years. No such age-dependent effect of depressive symptoms was seen in the general population (Figure 2). The age-dependent pattern was confirmed in the log-minus-log-plot used to evaluate the PH assumption in the Cox regression model (Supplemental material, Figure S1). The age-dependent pattern in the AAA group remained in the analyses adjusted for potential confounders, as illustrated in the predicted survival plots based on the adjusted Cox-PH regression models stratified on HADS-D and AAA to allow for deviation from the PH assumption (Figure 4). HR values are not calculated for stratification variables in the Cox PH model, and age-specific HR values for HADS-D (≥8 vs. >8) in subgroups defined by AAA (no, yes) can thus not be reported. A low number of deaths, as well as a low number of individuals at risk in the subgroup with both AAA and depressive symptoms below the age of 70 years (Figure 2), precluded an age-specific analysis.

The log-minus-log plot (Supplemental material, Figure S1) also indicated deviation from the PH assumption for BMI category. Additional analyses using BMI category as a stratification variable in the Cox regression model did not notably impact the HR values for our main study variables (AAA and HADS-D).

For peer review only

Table 1. Demographic and clinical characteristics at start of follow-up, total and by AAA

Characteristics	Total population n (%)	AAA Yes n (%)	AAA No n (%)	P-value
**Total**	36616 (100)	583 (100)	36033 (100)	
**Sex**				<0.001
Men	17883 (48.8)	432 (74.1)	17451 (48.4)	
Women	18733 (51.2)	151 (25.9)	18582 (51.6)	
**Age at measured HADS-D**				<0.001
<60	20843 (56.9)			
60-69	9550 (26.1)			
70-79	5254 (14.3)			
≥80	969 (2.4)			
**HADS-D**				<0.001
<8	31784 (86.3)	477 (81.8)	31307 (86.9)	
≥8	4832 (13.2)	106 (18.2)	4726 (13.1)	
**Smoking**				<0.001
Never	12890 (35.9)	53 (9.2)	12837 (36.4)	
Past	12738 (35.5)	192 (33.5)	12546 (35.5)	
Current	10260 (28.6)	12546 (35.5)	9931 (28.1)	
**BMI**				0.26
<25	11198 (30.8)	177 (30.7)	11021 (30.8)	
25-29	17349 (47.8)	280 (48.5)	17069 (47.7)	
30-35	6107 (16.8)	103 (17.8)	6004 (16.8)	
≥35	1679 (4.6)	17 (3.0)	1662 (4.7)	
**CHD**	3347 (9.2)	423 (27.4)	160 (8.9)	<0.001
**Hypertension**	21124 (57.7)	423 (72.5)	20701 (57.5)	<0.001
**Diabetes**	1746 (4.8)	23 (4.0)	1723 (4.8)	0.09
**Civil status**				0.35
Cohabiting	26650 (72.9)	443 (76.0)	26207 (72.8)	
Living alone	9913 (27.1)	140 (24.0)	9773 (27.2)	

AAA = Abdominal Aortic Aneurysm, HADS = Hospital Anxiety and Depression Scale, D = depression subscale, BMI = Body Mass Index, CHD = coronary heart disease
Missing data among those with valid HADS-D (n = 36616): Smoking, n = 728 (2.6%) BMI, n = 283 (0.8%), CHD, n = 23 (0.06%), systolic and diastolic blood pressure, n = 154 (0.4%), hypertension, n = 9 (0.02%), diabetes, n = 84 (0.2%), civil status, n = 53 (0.14%).
Missing data on HADS-D, n = 5259 (12.6%).

For peer review only

Table 2 Hazard ratio (HR with 95 % CI) of death for AAA (yes vs. no) and for depressive symptoms (HADS-D ≥ 8 vs < 8) in subgroups defined by AAA (yes,no)*

	No. of deahts	Univariate HR (95% CI)*	Multivariate HR (95% CI)**
**AAA**			
No	9078	1.00	1.00
Yes	350	3.39 (3.01-3.78)	2.66 (2.39-2.97)
**HADS-D**			
Individuals with AAA			
<8	283	1.00	1.00
≥8	67	1.28 (0.98-1.69)	1.15 (0.88-1.51)
Individuals without AAA			
<8	7430	1.00	1.00
≥8	1648	1.28 (1.21-1.36)	1.23 (1.17-1.30)
**P-value, interaction**		0.90	0.62

Depressive symptoms were defined as a HADS-D score ≥8.
AAA = Abdominal Aortic Aneurysm, HR = Hazard ratio, CI = Confidence Interval, HADS= Hospital Anxiety and Depression Scale.
*Based on Cox PH regression model with attained age as time scale.
**Additional adjustment for sex, coronary heart disease, diabetes mellitus, body mass index-category, smoking, hypertension, civil status. HR for AAA also adjusted for HADS-D.
Complete case analyses: Number of individuals, n= 34464.

For peer review only

For peer review only - http://bmjopen.bmj.com/site/about/guidelines.xhtml

DISCUSSION

In this large, population-based prospective study, the overall risk of death was more than twice as high in individuals with AAA compared to a general population at the same age and same risk profile. There was no clear adverse effect of depressive symptoms on all-cause mortality in individuals diagnosed with AAA after adjustment for potential confounders, whereas a negative prognostic effect remained in the general population. At ages <70 years, however, there was a tendency towards a more pronounced adverse effect of depressive symptoms in individuals with AAA. This finding was uncertain due to the low number of deaths before the age of 70 years.

The finding of a substantially higher mortality in individuals with vs. without AAA is consistent with a recent study reporting markedly lower overall long-term survival in patients undergoing successful AAA repair as compared to age-and sex-adjusted mortality rates in the general population.[27] An unfavorable co-morbidity profile commonly seen in AAA patients, including other cardiovascular diseases, as well as smoking, is probably a contributing factor to the high crude mortality rates in individuals with AAA.[28] In our study, however, the relative risk estimate remained high also after adjusting for factors associated with both AAA and increased mortality, such as sex, coronary heart disease, diabetes and smoking. We are not aware of any previous studies that have compared mortality in individuals with AAA with a general population of the same age and also same level of other risk factors. Most previous studies have almost exclusively been restricted to patients that have undergone surgery for AAA or patients deemed unfit for surgery.[27, 29-31] The present study included both surgically untreated and treated AAA patients, but we did not distinguish between these subgroups in the analyses due to lack of information on this issue.

For peer review only - http://bmjopen.bmj.com/site/about/guidelines.xhtml

To our knowledge, no previous studies have examined whether depressive symptoms on mortality differ in patients with AAA compared to a general population. The results from the present study did not confirm our initial hypothesis that a negative prognostic impact of depressive symptoms may be stronger in individuals with AAA compared to a general population. On the contrary, there was no clear association between depressive symptoms and mortality in the AAA group. The lack of association could relate to the low number of individuals with both AAA and depressive symptoms. However, we did observe a slightly more pronounced adverse effect of depressive symptoms in individuals diagnosed with AAA before the age of 70 years, whereas no such age-dependent effect was seen in the general population. This pattern may relate to the fact that some risk factors for AAA, such as a high number of pack years (smoking) and unfavorable heredity patterns have been reported to be more common in young compared to older AAA patients.[32] We did adjust for smoking habits, whereas data on heritability was not available. Due to the low number of individuals in these age groups, these results must be interpreted with caution and confirmed in other studies.

The biological mechanisms behind the increased mortality rates in individuals with AAA are yet to be clarified. Inflammation seems to be an important part of both the AAA pathogenesis and growth,[33, 34] which may relate to impact from noxious stimuli such as cigarette smoke, low density lipoprotein cholesterol and hypertension.[35] With increasing age, competent repair cells are not always available, artery repair fails and the inflammatory state increases.[1] Previous studies have shown that depression is associated with low-grade chronic inflammation.[1, 15, 36] Moreover, a negative impact from inflammatory processes on mortality in patients with CVDs and concurrent depression has been suggested.[37] Our

For peer review only - http://bmjopen.bmj.com/site/about/guidelines.xhtml

finding indicating that depressive symptoms might worsen the prognosis in younger, but not in older individuals diagnosed with AAA might be somewhat contradictory. On the other hand, it is possible that individuals who develop AAA at a younger age have faster aneurysm growth that is more susceptible to a growth-enhancing effect from depression, potentially mediated by inflammation.

Strengths and limitations

A major strength of our study is that the results are based on data from a large, population-based, prospective study. The HUNT population is a validated and well-defined homogenous cohort that represents the general population in Norway. Data on risk factors were assessed at the individual level for all study participants, enabling tight adjustment for age (1- year intervals, time scale in the survival analyses), sex and other potential confounders when estimating HR values. Repeated measurements of the exposure factors made it possible to update the risk set by use of time-varying covariates. This analytic approach reduces potential misclassification bias in the risk estimates. However, not all the individuals included in our study had participated in HUNT more than once, which is a limitation. The amount of missing data for the covariates was low. All AAA cases were verified based on a certified hospital diagnosis and dates of death were obtained from national registers with compulsory recordings. It is, however, likely that there was some underdiagnosis of AAA cases. The HUNT study does not include ultrasound examinations of the abdominal aorta. The observed 1.6% crude prevalence of AAA in our study is, however, in line with the expected rate at the time.[38] Unless a potential underdiagnosis of AAA differ by value of HADS-D, the risk estimates will not be biased. Some limitations must be considered. Most data on covariates were self-reported, including symptoms of depression, cardiovascular comorbidities and

For peer review only

For peer review only - http://bmjopen.bmj.com/site/about/guidelines.xhtml

smoking, potentially leading to information bias. Symptoms of depression were assessed through self-reported questionnaires and not by a psychiatric diagnostic interview. We had no information about antidepressant use. The low number of individuals with severe depressive symptoms in our study population could be a result from recruitment bias. Individuals with severe depression might not adhere to screening programs or attend population-based studies to the same extent as non-depressed individuals. However, such recruitment bias would likely bias our results towards the null hypothesis.

CONCLUSION

In this population-based prospective study there was no significant prognostic impact of depressive symptoms on mortality in individuals with AAA, whereas an increased risk was seen in the general population. Our results revealed a hitherto unreported higher all-cause mortality in individuals with AAA compared to a general population of the same age and same risk profile. More studies with larger AAA cohorts are needed to explore the relation between depression, AAA and mortality, and a potential heterogeneity across age groups.

Acknowledgements

The Trøndelag Health Study (The HUNT Study) is a collaboration between HUNT Research Centre, (Faculty of Medicine and Health Sciences, NTNU, Norwegian University of Science and Technology), Trøndelag County Council, Central Norway Regional Health Authority, and the Norwegian Institute of Public Health.

For peer review only

Contributorship

LN contributed to study conception and design, data and statistical analysis, interpretation of study findings and drafting and critical review of the manuscript. RH contributed to study conception and design, interpretation of study findings and drafting and critical review of the manuscript.

GA contributed to study conception, and statistical methods and analysis, interpretation of study findings and critical review of the manuscript. EM contributed to study conception and design and critical review of the manuscript. MS contributed to study conception and design, interpretation of study findings and drafting and critical review of the manuscript.

Data availability statement

No data are available

The Trøndelag Health Study (HUNT) has invited persons aged 13 - 100 years to four surveys between 1984 and 2019. Comprehensive data from more than 140.000 persons having participated at least once and biological material from 78.000 persons are collected. The data are stored in HUNT databank and biological material in HUNT biobank. HUNT Research Centre has permission from the Norwegian Data Inspectorate to store and handle these data. The key identification in the data base is the personal identification number given to all Norwegians at birth or immigration, whilst de-identified data are sent to researchers upon approval of a research protocol by the Regional Ethical Committee and HUNT Research Centre. To protect participants' privacy, HUNT Research Centre aims to limit storage of data outside HUNT databank, and cannot deposit data in open repositories. HUNT databank has precise information on all data exported to different projects and are able to reproduce these on request. There are no restrictions regarding data export given approval of

For peer review only

applications to HUNT Research Centre. For more information see: https://eur01.safelinks.protection.outlook.com/?url=http%3A%2F%2Fwww.ntnu.edu%2Fhunt%2Fdata&data=04%7C01%7Cmalin.stenman%40ki.se%7C1bc54c35ed5f4ce79e0508d8a7616b52%7Cbff7eef1cf4b4f32be3da1dda043c05d%7C0%7C0%7C637443384157146991%7CUnknown%7CTWFpbGZsb3d8eyJWIjoiMC4wLjAwMDAiLCJQIjoiV2luMzIiLCJBTiI6Ik1haWwiLCJXVCI6Mn0%3D%7C1000&sdata=GwGqQpTG4u%2FoslrgO43q64XHIpvyeEMTSCo%2ByrTn7i4%3D&reserved=0

Competing interests

None declared

Funding

This research has been supported by the Swedish Heart-Lung Foundation (Hultgren) grant number: 20190553 and 20180506.

Patient consent for publication

Not required

Ethics approval

The study was approved by the HUNT Study board of directors and the Regional Ethics Committee (2014/175/REK12 Midt-Norge).

Disclosures

The authors of this manuscript have no potential conflicts of interest.

For peer review only - http://bmjopen.bmj.com/site/about/guidelines.xhtml

REFERENCES

1. Sakalihasan N, Michel JB, Katsargyris A, et al. Abdominal aortic aneurysms. *Nat Rev Dis Primers* 2018;4:34.
2. Hare DL, Toukhsati SR, Johansson P, et al. Depression and cardiovascular disease: a clinical review. *Eur Heart J* 2014;35:1365-72.
3. Bath MF, Sidloff D, Saratzis A, et al. Impact of abdominal aortic aneurysm screening on quality of life. *Br J Surg* 2018;105:203-08.
4. Wanhainen A, Hultgren R, Linne A, et al. Outcome of the Swedish Nationwide Abdominal Aortic Aneurysm Screening Program. *Circulation* 2016;134:1141-48.
5. Norman PE, Jamrozik K, Lawrence-Brown MM, et al. Population based randomised controlled trial on impact of screening on mortality from abdominal aortic aneurysm. *Bmj* 2004;329:1259.
6. Ashton HA, Buxton MJ, Day NE, et al. The Multicentre Aneurysm Screening Study (MASS) into the effect of abdominal aortic aneurysm screening on mortality in men: a randomised controlled trial. *Lancet* 2002;360:1531-9.
7. Lindholt JS, Juul S, Fasting H, et al. Screening for abdominal aortic aneurysms: single centre randomised controlled trial. *Bmj* 2005;330:750.
8. Ali MU, Fitzpatrick-Lewis D, Miller J, et al. Screening for abdominal aortic aneurysm in asymptomatic adults. *J Vasc Surg* 2016;64:1855-68.
9. Timmers TK, van Herwaarden JA, de Borst GJ, et al. Long-term survival and quality of life after open abdominal aortic aneurysm repair. *World J Surg* 2013;37:2957-64.
10. de Bruin JL, Baas AF, Heymans MW, et al. Statin therapy is associated with improved survival after endovascular and open aneurysm repair. *J Vasc Surg* 2014;59:39-44.e1.
11. Bath MF, Gokani VJ, Sidloff DA, et al. Systematic review of cardiovascular disease and cardiovascular death in patients with a small abdominal aortic aneurysm. *Br J Surg* 2015;102:866-72.
12. Mykletun A, Bjerkeset O, Overland S, et al. Levels of anxiety and depression as predictors of mortality: the HUNT study. *Br J Psychiatry* 2009;195:118-25.
13. Harshfield EL, Pennells L, Schwartz JE, et al. Association Between Depressive Symptoms and Incident Cardiovascular Diseases. *Jama* 2020;324:2396-405.
14. Stenman M, Holzmann MJ, Sartipy U. Relation of major depression to survival after coronary artery bypass grafting. *Am J Cardiol* 2014;114:698-703.
15. Nemeroff CB, Goldschmidt-Clermont PJ. Heartache and heartbreak--the link between depression and cardiovascular disease. *Nat Rev Cardiol* 2012;9:526-39.
16. Daskalopoulou M, George J, Walters K, et al. Depression as a Risk Factor for the Initial Presentation of Twelve Cardiac, Cerebrovascular, and Peripheral Arterial Diseases: Data Linkage Study of 1.9 Million Women and Men. *PLoS One* 2016;11:e0153838.
17. Nyronning LA, Stenman M, Hultgren R, et al. Symptoms of Depression and Risk of Abdominal Aortic Aneurysm: A HUNT Study. *J Am Heart Assoc* 2019;8:e012535.
18. Pan A, Sun Q, Okereke OI, et al. Depression and risk of stroke morbidity and mortality: a meta-analysis and systematic review. *Jama* 2011;306:1241-9.
19. Sakalihasan N, Van Damme H, Gomez P, et al. Positron emission tomography (PET) evaluation of abdominal aortic aneurysm (AAA). *Eur J Vasc Endovasc Surg* 2002;23:431-6.

For peer review only

For peer review only - http://bmjopen.bmj.com/site/about/guidelines.xhtml

20. Dale MA, Ruhlman MK, Baxter BT. Inflammatory cell phenotypes in AAAs: their role and potential as targets for therapy. *Arterioscler Thromb Vasc Biol* 2015;35:1746-55.
21. Almeida OP, Ford AH, Hankey GJ, et al. Depression, antidepressants and the risk of cardiovascular events and death in older men. *Maturitas* 2019;128:4-9.
22. Cuijpers P, Vogelzangs N, Twisk J, et al. Comprehensive meta-analysis of excess mortality in depression in the general community versus patients with specific illnesses. *Am J Psychiatry* 2014;171:453-62.
23. Letnes JM, Torske MO, Hilt B, et al. Symptoms of depression and all-cause mortality in farmers, a cohort study: the HUNT study, Norway. *BMJ Open* 2016;6:e010783.
24. Krokstad S, Langhammer A, Hveem K, et al. Cohort Profile: the HUNT Study, Norway. *Int J Epidemiol* 2013;42:968-77.
25. Bjelland I, Dahl AA, Haug TT, et al. The validity of the Hospital Anxiety and Depression Scale. An updated literature review. *J Psychosom Res* 2002;52:69-77.
26. Myrtveit SM, Ariansen AM, Wilhelmsen I, et al. A population based validation study of self-reported pensions and benefits: the Nord-Trondelag health study (HUNT). *BMC Res Notes* 2013;6:27.
27. Bulder RMA, Talvitie M, Bastiaannet E, et al. Long-term Prognosis After Elective AAA Repair is Poor in Women and Men: The Challenges Remain. *Ann Surg* 2020.
28. Toghill BJ, Saratzis A, Bown MJ. Abdominal aortic aneurysm-an independent disease to atherosclerosis? *Cardiovasc Pathol* 2017;27:71-75.
29. Whittaker JD, Meecham L, Summerour V, et al. Outcome after Turndown for Elective Abdominal Aortic Aneurysm Surgery. *Eur J Vasc Endovasc Surg* 2017;54:579-86.
30. Rueda-Ochoa OL, van Bakel P, Hoeks SE, et al. Survival After Uncomplicated EVAR in Octogenarians is Similar to the General Population of Octogenarians Without an Abdominal Aortic Aneurysm. *Eur J Vasc Endovasc Surg* 2020;59:740-47.
31. Norman PE, Semmens JB, Lawrence-Brown MM, et al. Long term relative survival after surgery for abdominal aortic aneurysm in western Australia: population based study. *Bmj* 1998;317:852-6.
32. Howard DP, Banerjee A, Fairhead JF, et al. Age-specific incidence, risk factors and outcome of acute abdominal aortic aneurysms in a defined population. *Br J Surg* 2015;102:907-15.
33. Kuivaniemi H, Ryer EJ, Elmore JR, et al. Understanding the pathogenesis of abdominal aortic aneurysms. *Expert Rev Cardiovasc Ther* 2015;13:975-87.
34. Eagleton MJ. Inflammation in abdominal aortic aneurysms: cellular infiltrate and cytokine profiles. *Vascular* 2012;20:278-83.
35. Rocha VZ, Libby P. Obesity, inflammation, and atherosclerosis. *Nat Rev Cardiol* 2009;6:399-409.
36. Raedler TJ. Inflammatory mechanisms in major depressive disorder. *Curr Opin Psychiatry* 2011;24:519-25.
37. Celano CM, Huffman JC. Depression and cardiac disease: a review. *Cardiol Rev* 2011;19:130-42.
38. Ulug P, Powell JT, Sweeting MJ, et al. Meta-analysis of the current prevalence of screen-detected abdominal aortic aneurysm in women. *Br J Surg* 2016;103:1097-104.

For peer review only

Figure legends

Figure 1.
Kaplan-Meier failure plot and corresponding Nelson-Aalen cumulative hazard estimates in subgroups defined by AAA (no, yes)

Figure 2.
Kaplan-Meier failure plot and corresponding Nelson-Aalen cumulative hazard estimates in subgroups defined by HADS-D (< 8 and ≥8) and AAA (no, yes).

Figure 3.
HR values with 95 % CI for the combined categories of AAA (no, yes) and HADS-D, using a common reference group (no AAA, HADS-D < 8). The results are based on the adjusted Cox PH regression model.
AAA = Abdominal Aortic Aneurysm, HADS = Hospital Anxiety and Depression Scale.

Figure 4.
Predicted survival plots based on a Cox PH regression model stratified for HADS-D and AAA (combined categories) to allow for an age-dependent effect. Curves are calculated for reference categories of adjustment factors (sex, BMI category, smoking, CHD, diabetes mellitus, hypertension and civil status). Complete case analyses.

For peer review only

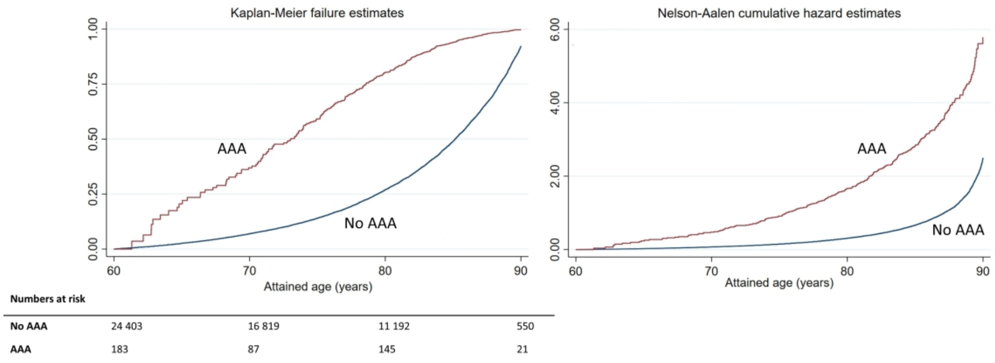

Kaplan-Meier failure plot and corresponding Nelson-Aalen cumulative hazard estimates in subgroups defined by AAA (no, yes)

338x190mm (300 x 300 DPI)

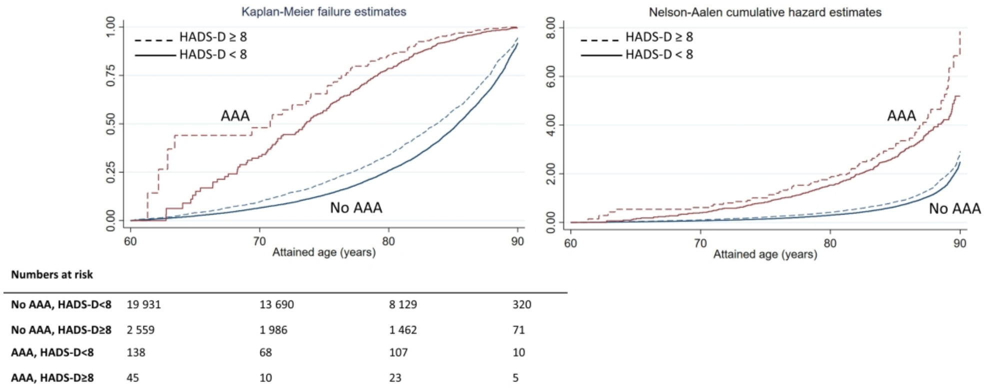

Kaplan-Meier failure plot and corresponding Nelson-Aalen cumulative hazard estimates in subgroups defined by HADS-D (< 8 and ≥8) and AAA (no, yes).

338x190mm (300 x 300 DPI)

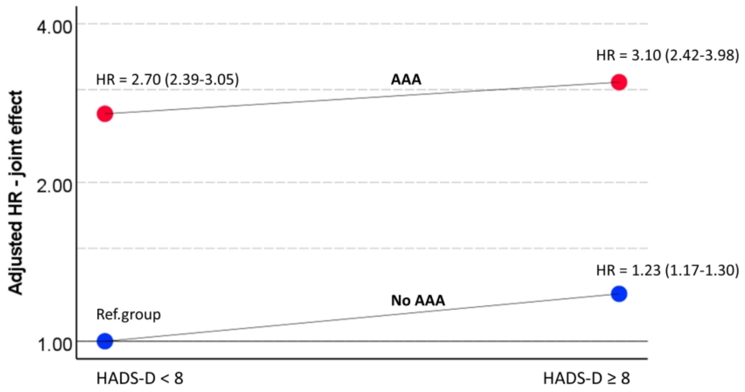

HR values with 95 % CI for the combined categories of AAA (no, yes) and HADS-D, using a common reference group (no AAA, HADS-D < 8). The results are based on the adjusted Cox PH regression model.
AAA = Abdominal Aortic Aneurysm, HADS = Hospital Anxiety and Depression Scale.

338x190mm (300 x 300 DPI)

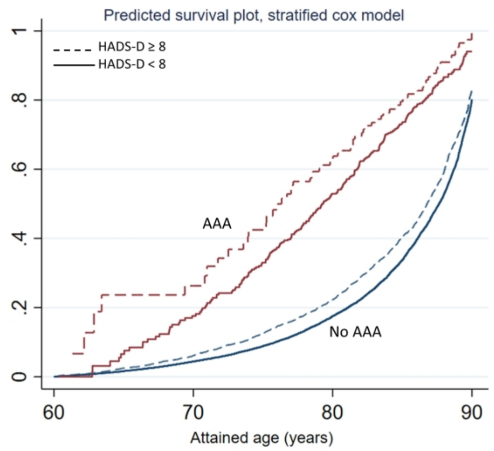

Predicted survival plots based on a Cox PH regression model stratified for HADS-D and AAA (combined categories) to allow for an age-dependent effect. Curves are calculated for reference categories of adjustment factors (sex, BMI category, smoking, CHD, diabetes mellitus, hypertension and civil status). Complete case analyses.

338x190mm (300 x 300 DPI)

Supplemental material

The prognostic impact of depressive symptoms on all-cause mortality in individuals with abdominal aortic aneurysm and in the general population. A population-based prospective HUNT Study in Norway

Linn Å. Nyrønning[1,2] Rebecka Hultgren[3,4] Grethe Albrektsen[5,6], Erney Mattsson[1,2]

Malin Stenman[3,7]

[1]Department of Surgery, Vascular Surgery, St Olavs Hospital, Trondheim, Norway,

[2]Department of Circulation and Medical Imaging, NTNU, Trondheim, Norway,

[3]Department of Molecular Medicine and Surgery, Karolinska Institutet, Stockholm, Sweden,

[4]Department of Vascular Surgery, Karolinska University Hospital, Stockholm, Sweden,

[5]Department of Public Health and Nursing, NTNU, Trondheim, Norway,

[6]Department of clinical and molecular medicine, NTNU, Trondheim, Norway,

[7]Perioperative Medicine and Intensive Care Function, Karolinska University Hospital, Stockholm, Sweden

Corresponding author:

Malin Stenman

Perioperative Medicine and Intensive Care Function

Karolinska University Hospital / Karolinska Institutet

SE-171 76 Stockholm, Sweden

E-mail: Malin.Stenman@ki.se

For peer review only

Figure S1. "Log - log plot" to test the proportional hazards assumption. If the plotted lines are reasonably parallel, the proportional hazards assumption has not been violated.

For peer review only

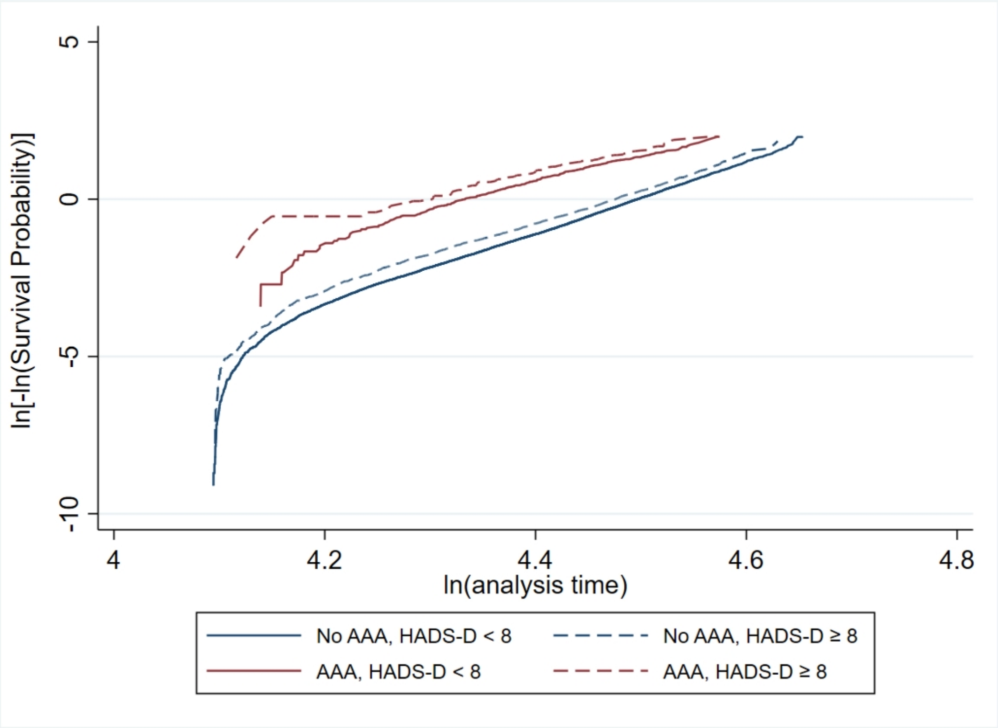

Figure S1. "Log - log plot" to test the proportional hazards assumption. If the plotted lines are reasonably parallel, the proportional hazards assumption has not been violated.

415x302mm (300 x 300 DPI)

STROBE 2007 (v4) Statement—Checklist of items that should be included in reports of *cohort studies*

Section/Topic	Item #	Recommendation	Reported on page #
**Title and abstract**	1	(*a*) Indicate the study's design with a commonly used term in the title or the abstract	1
		(*b*) Provide in the abstract an informative and balanced summary of what was done and what was found	2
**Introduction**			
Background/rationale	2	Explain the scientific background and rationale for the investigation being reported	4-5
Objectives	3	State specific objectives, including any prespecified hypotheses	5
**Methods**			
Study design	4	Present key elements of study design early in the paper	5
Setting	5	Describe the setting, locations, and relevant dates, including periods of recruitment, exposure, follow-up, and data collection	6-8
Participants	6	(*a*) Give the eligibility criteria, and the sources and methods of selection of participants. Describe methods of follow-up	6
		(*b*) For matched studies, give matching criteria and number of exposed and unexposed	6
Variables	7	Clearly define all outcomes, exposures, predictors, potential confounders, and effect modifiers. Give diagnostic criteria, if applicable	6-9
Data sources/ measurement	8*	For each variable of interest, give sources of data and details of methods of assessment (measurement). Describe comparability of assessment methods if there is more than one group	7-8
Bias	9	Describe any efforts to address potential sources of bias	9
Study size	10	Explain how the study size was arrived at	6
Quantitative variables	11	Explain how quantitative variables were handled in the analyses. If applicable, describe which groupings were chosen and why	7-9
Statistical methods	12	(*a*) Describe all statistical methods, including those used to control for confounding	8-9
		(*b*) Describe any methods used to examine subgroups and interactions	9
		(*c*) Explain how missing data were addressed	9
		(*d*) If applicable, explain how loss to follow-up was addressed	
		(*e*) Describe any sensitivity analyses	9
**Results**			

For peer review only

Participants	13*	(a) Report numbers of individuals at each stage of study—eg numbers potentially eligible, examined for eligibility, confirmed eligible, included in the study, completing follow-up, and analysed	6,7,11
		(b) Give reasons for non-participation at each stage	
		(c) Consider use of a flow diagram	
Descriptive data	14*	(a) Give characteristics of study participants (eg demographic, clinical, social) and information on exposures and potential confounders	11
		(b) Indicate number of participants with missing data for each variable of interest	14
		(c) Summarise follow-up time (eg, average and total amount)	7
Outcome data	15*	Report numbers of outcome events or summary measures over time	11-12
Main results	16	(*a*) Give unadjusted estimates and, if applicable, confounder-adjusted estimates and their precision (eg, 95% confidence interval). Make clear which confounders were adjusted for and why they were included	11-13
		(*b*) Report category boundaries when continuous variables were categorized	8, 14
		(*c*) If relevant, consider translating estimates of relative risk into absolute risk for a meaningful time period	
Other analyses	17	Report other analyses done—eg analyses of subgroups and interactions, and sensitivity analyses	11-13
**Discussion**			
Key results	18	Summarise key results with reference to study objectives	16
**Limitations**			16-19
Interpretation	20	Give a cautious overall interpretation of results considering objectives, limitations, multiplicity of analyses, results from similar studies, and other relevant evidence	19
Generalisability	21	Discuss the generalisability (external validity) of the study results	19
**Other information**			
Funding	22	Give the source of funding and the role of the funders for the present study and, if applicable, for the original study on which the present article is based	21

*Give information separately for cases and controls in case-control studies and, if applicable, for exposed and unexposed groups in cohort and cross-sectional studies.

Note: An Explanation and Elaboration article discusses each checklist item and gives methodological background and published examples of transparent reporting. The STROBE checklist is best used in conjunction with this article (freely available on the Web sites of PLoS Medicine at http://www.plosmedicine.org/, Annals of Internal Medicine at http://www.annals.org/, and Epidemiology at http://www.epidem.com/). Information on the STROBE Initiative is available at www.strobe-statement.org.

For peer review only

BMJ Open

The prognostic impact of depressive symptoms on all-cause mortality in individuals with abdominal aortic aneurysm and in the general population. A population-based prospective HUNT study in Norway

Journal:	*BMJ Open*
Manuscript ID	bmjopen-2021-049055.R3
Article Type:	Original research
Date Submitted by the Author:	29-Oct-2021
Complete List of Authors:	Nyrønning, Linn Å.; St Olavs Hospital Universitetssykehuset i Trondheim, Department of Surgery, Vascular Surgery; NTNU Department of Circulation and Medical Imaging<br>Hultgren, Rebecka; Karolinska Institute, Deparment of molecular medicine and surgery; Karolinska Universitetssjukhuset, Department of Vascular Surgery<br>Albrektsen, Grethe; Norges teknisk-naturvitenskapelige universitet, Department of public health and general practice<br>Mattsson, Erney; St Olavs Hospital Universitetssykehuset i Trondheim; NTNU Department of Circulation and Medical Imaging<br>Stenman, Malin ; Karolinska Institutet, Department of Molecular Medicine and Surgery; Karolinska University Hospital, Department of Molecular Medicine and Surgery
**Primary Subject Heading**:	Epidemiology
Secondary Subject Heading:	Epidemiology, Cardiovascular medicine
Keywords:	EPIDEMIOLOGY, Vascular medicine < INTERNAL MEDICINE, Vascular surgery < SURGERY, VASCULAR SURGERY

SCHOLARONE™
Manuscripts

I, the Submitting Author has the right to grant and does grant on behalf of all authors of the Work (as defined in the below author licence), an exclusive licence and/or a non-exclusive licence for contributions from authors who are: i) UK Crown employees; ii) where BMJ has agreed a CC-BY licence shall apply, and/or iii) in accordance with the terms applicable for US Federal Government officers or employees acting as part of their official duties; on a worldwide, perpetual, irrevocable, royalty-free basis to BMJ Publishing Group Ltd ("BMJ") its licensees and where the relevant Journal is co-owned by BMJ to the co-owners of the Journal, to publish the Work in this journal and any other BMJ products and to exploit all rights, as set out in our licence.

The Submitting Author accepts and understands that any supply made under these terms is made by BMJ to the Submitting Author unless you are acting as an employee on behalf of your employer or a postgraduate student of an affiliated institution which is paying any applicable article publishing charge ("APC") for Open Access articles. Where the Submitting Author wishes to make the Work available on an Open Access basis (and intends to pay the relevant APC), the terms of reuse of such Open Access shall be governed by a Creative Commons licence – details of these licences and which Creative Commons licence will apply to this Work are set out in our licence referred to above.

Other than as permitted in any relevant BMJ Author's Self Archiving Policies, I confirm this Work has not been accepted for publication elsewhere, is not being considered for publication elsewhere and does not duplicate material already published. I confirm all authors consent to publication of this Work and authorise the granting of this licence.

For peer review only

The prognostic impact of depressive symptoms on all-cause mortality in individuals with abdominal aortic aneurysm and in the general population. A population-based prospective HUNT study in Norway

Linn Å. Nyrønning[1,2] Rebecka Hultgren[3,4] Grethe Albrektsen[5,6], Erney Mattsson[1,2] Malin Stenman[3,7]

[1]Department of Surgery, Vascular Surgery, St Olavs Hospital, Trondheim, Norway,

[2]Department of Circulation and Medical Imaging, NTNU, Trondheim, Norway,

[3]Department of Molecular Medicine and Surgery, Karolinska Institutet, Stockholm, Sweden,

[4]Department of Vascular Surgery, Karolinska University Hospital, Stockholm, Sweden,

[5]Department of Public Health and Nursing, NTNU, Trondheim, Norway,

[6]Department of clinical and molecular medicine, NTNU, Trondheim, Norway,

[7]Perioperative Medicine and Intensive Care Function, Karolinska University Hospital, Stockholm, Sweden

Corresponding author:

Malin Stenman

Perioperative Medicine and Intensive Care Function

Karolinska University Hospital / Karolinska Institutet

SE-171 76 Stockholm, Sweden

E-mail: Malin.Stenman@ki.se

Word count: 3549

For peer review only

ABSTRACT

Background Abdominal aortic aneurysm (AAA) is a potentially life-threatening disease but the high mortality rate is linked to high age and comorbidity pattern. Depression is associated with increased mortality in the general population and individuals with cardiovascular diseases, but this is sparsely studied for AAA. The aim was to examine the prognostic impact of depressive symptoms on all-cause mortality in individuals with AAA and compare with findings in a general population of the same age and risk profile.

Methods Population-based prospective study including 36616 participants (52.1% women) from the Trøndelag Health Study (HUNT) in Norway. A total of 9428 individuals died during a median follow-up of 10 years at ages 60-90 years. Depressive symptoms were defined by a Hospital Anxiety and Depression (HADS-D) score ≥ 8. Data on AAA diagnoses and death were obtained from medical records and national registers. Hazard ratios (HR) from Cox proportional hazard regression models are reported.

Results A total of 4832 (13.2%) individuals reported depressive symptoms, whereas 583 (1.6%) AAAs were identified. The adjusted hazard of death was 2.66 times higher in persons with AAA compared to the general population (95%CI 2.39-2.97). Overall, there was no significant adverse effect of depressive symptoms in individuals with AAA (HR=1.15;95% CI 0.88-1.51), whereas an increased risk was seen in the general population (HR=1.23;95% CI 1.17-1.30).

Conclusion The overall risk of death was considerably higher in individuals with AAA compared to a general population of the same age and risk profile. Depressive symptoms did not significantly influence the risk of death in the AAA group.

For peer review only

Strengths and limitations of this study

- The results from this study are based on data from a large and population-based prospective study in Norway.
- The general population served as comparison group when evaluating the prognostic impact of depressive symptoms on all-cause mortality in individuals diagnosed with abdominal aortic aneurysm.
- The risk estimates were adjusted for potential confounders, such as age, sex, smoking, coronary heart disease, diabetes, BMI, hypertension and civil status.
- All AAA cases were verified based on a certified hospital diagnosis, but due to the lack of ultrasound measurement in HUNT, the prevalence of AAA in the general population may have been underestimated.
- Data on depressive symptoms and comorbidity patterns were self-reported.

For peer review only

INTRODUCTION

Abdominal aortic aneurysm (AAA) is a localized widening of the abdominal aorta that often is asymptomatic and undiagnosed.[1] The most feared complication of an AAA is rupture, a life-threatening bleeding associated with fatal outcome if untreated.[1] To prevent deaths from a ruptured AAA, an early diagnosis and treatment in due time is essential. AAA is in general a late onset disease, that is associated with an unfavorable co-morbidity pattern and high mortality rates. [2, 3] Screening of men aged 65 years or older seems to reduce the risk of deaths related to AAA by up to 30 to 40 %.[4-7] whereas the effect on all-cause mortality is less clear.[8] Nonetheless, mortality rates in patients that have undergone surgery for AAA have been found to be higher than for patients with other cardiovascular diseases (CVDs). [9, 10] High mortality in patients diagnosed with AAA has been linked to high age and unfavorable comorbidity patters, including death from cardiovascular diseases and other smoking-related diseases. Many individuals diagnosed with AAA live with an asymptomatic aneurysm or undergo elective surgery, and eventually die from other causes.[11] Very few previous studies have compared the overall mortality in individuals diagnosed with AAA to a general population of the same age and with the same risk profile.

Depression is associated with increased mortality in the general population.[12] Moreover, depression is common in patients with cardiovascular disease [13] and is independently associated with increased cardiovascular morbidity and mortality.[2, 14, 15] In two recent large prospective, population-based studies, depression was associated with increased risk of AAA.[16, 17] AAA and depression have both been associated with low-grade inflammation.[12, 18] Hence, inflammation could be a common risk factor that may explain the observed association between AAA and depression. Alternatively, the inflammation

For peer review only

process evolves on the pathway between depression and the risk of AAA. Previous studies have suggested that inflammation may increase the risk of rupture by triggering a more rapid growth of the aneurysm.[19, 20] If depression, as a proxy for low grade inflammation, influences both the risk and progression of AAA development, an adverse prognostic impact of depression may be particularly pronounced in individuals with AAA. An adverse effect of depression on mortality rates in patients with other cardiovascular diseases has been established [12, 15, 18, 21-23], but few previous studies have examined the prognostic impact of depression in individuals with AAA. Moreover, we are not aware of any previous studies that have compared with findings in a general population.

The main aim of the present study was to examine whether the prognostic impact of depressive symptoms on all-cause mortality in individuals diagnosed with AAA differed from the established adverse effect in the general population. Moreover, we investigate whether there was an overall difference in all-cause mortality between individuals diagnosed with AAA and the general population of the same age and with the same risk profile.

METHODS

This is a prospective population-based cohort study, based on data obtained from the Norwegian Trøndelag Health Study (HUNT). The study has been approved by the HUNT Study board of directors and the Regional Ethics Committee (2014/175/REK12 Midt-Norge), and was conducted in accordance with the Helsinki Declaration. The study was performed following the STROBE checklist for cohort studies (supplemental material).

For peer review only - http://bmjopen.bmj.com/site/about/guidelines.xhtml

The HUNT Study

HUNT is a large and ongoing multiphase population-based health study of people aged 20 years or older conducted in the county of Nord-Trøndelag in central Norway.[24] The databank contains extensive information about a wide variety of demographics and health-related topics. Data on depressive symptom was introduced in the second HUNT survey (HUNT2).

Study population

The present study population includes 36616 individuals (52.1 % women) who participated in HUNT2 and/or HUNT3 and had available data in HUNT on depressive symptoms. Median age at the first participation in HUNT was 58.6 years (range 40.6-89.8 years). The unique personal identification number was used to link data on AAA diagnoses (ICD-9/10) and death with data from the HUNT study.

Patient and Public Involvement

This study has no direct contact with patients or the public. The study utilize de-identified data from the HUNT study.

Primary outcome and follow-up period

The primary outcome in this study was all-cause mortality. The Norwegian Cause of Death Registry was used to acquire information on dates of death. Each individual was considered to be at risk of death from the age of 60 years, or from the date of first HUNT participation at a higher age, until date of death or, reaching the age of 90 years, or closing date of the study

For peer review only - http://bmjopen.bmj.com/site/about/guidelines.xhtml

(December 31, 2014). The lower and upper age limits were defined due to few deaths before the age of 60 years and few individuals with AAA at risk above the age of 90 years. Median age at start of follow-up was 60 years (range 60.0- 89.8 years) and median follow-up time was 10 years (1.1 days to 20 years). At the end of the study period, the median age was 73 years (range 60-90 years). Among the 36616 individuals included in the study (51.2 % women), 9428 (44.0 % women) died during follow-up.

Ascertainment of AAA

Information on the diagnosis of AAA in the period 1995 through 2014 was obtained from local hospital registers (in-hospital and outpatient). An indicator variable for whether or not a person had been diagnosed with AAA was defined according to codes 441.3-4 and I71.3-4 in the ninth and tenth editions of the International Classification of Diseases (ICD 9/10 codes). All AAA diagnoses were manually verified using patient charts.

Assessment of depressive symptoms

The Hospital Anxiety and Depression Scale (HADS) was used to assess the degree of depressive symptoms. HADS is a self-report questionnaire developed to evaluate patients in somatic care and aims to measure levels of anxiety and depression symptoms during the preceding week. The questionnaire consists of two separate subscales ranging from 0-21: the HADS Anxiety (HADS-A) and HADS Depression (HADS-D). Only the HADS-D score was used in the present study. A HADS-D score ≥ 8 was defined as having depressive symptoms. A cut-off at ≥ 8 (HADS-D ≥8) gives a specificity of 0.79 and a sensitivity of 0.83 compared with a clinical evaluation of depression.[25, 26]

For peer review only - http://bmjopen.bmj.com/site/about/guidelines.xhtml

Demographic and clinical factors

Information on potential confounders was assessed through responses on self-reported questionnaires and clinical examinations. Sex and civil status (categorized as living alone or with other) were assessed through questionnaires. Hypertension (yes, no) was defined as systolic blood pressure > 140 mmHg, diastolic blood pressure > 90 mmHg (average of second and third measurements) or use of antihypertensive medication (self-reported). Body mass index (BMI) was calculated as weight/height^2 (kg/m^2), and divided into four categories (<25, 25-30, 30-35, ≥35 kg/m^2). Coronary heart disease (CHD, yes, no) was defined as a composite variable of self-reported history of myocardial infarction or angina pectoris. Diabetes (yes, no), smoking status (never, past, current) were defined according to questionnaire responses.

Statistical analysis

Descriptive statistics at start of follow-up are presented as mean (standard deviation, SD), median (interquartile range), and/or as frequencies (%), according to nature of the data. Survival analyses with attained age as time scale in the follow-up period were carried out to evaluate the prognostic impact of HADS-D and AAA. The Kaplan-Meier approach was applied to calculate the expected proportion of non-survivors (proportion of deaths) at different ages for AAA (yes, no), and for combined categories of the HADS-D score (<8, ≥8) and the indicator variable for AAA (yes, no). The log-rank test was applied to compare the overall survival between the groups. In addition, the Nelson-Aalen estimate of the cumulative hazard function is presented. The shape of the curves in these plots is informative with respect to changes in hazard with increasing age in each exposure group. Age-adjusted and multivariable adjusted hazard ratios (HR) for death with 95 % confidence intervals (CI) were calculated as estimate of

For peer review only

For peer review only - http://bmjopen.bmj.com/site/about/guidelines.xhtml

relative risk of death in a Cox proportional hazard (PH) regression model. A multiplicative interaction term between HADS-D and AAA was added to evaluate whether the association between depressive symptoms and all-cause mortality differed for persons with and without AAA. HUNT participants without a diagnosis of AAA were considered representative for the general population. HR values adjusted for age only (time-scale), and with additional adjustment for sex, BMI category, smoking, CHD, diabetes mellitus, hypertension and civil status are presented. In the analyses of the overall association between AAA and mortality, we also adjusted for depressive symptoms. A backward stepwise procedure was applied to evaluate the individual contributions from single factors in the change from age-adjusted to multivariable adjusted HR-values, and to search for signs of potential overfitting and/or collinearity problems. The proportional hazard (PH) assumption for each covariate in the Cox PH regression model was visually evaluated in log-minus-log plots. Supplementary analyses stratified for adjustment factors that showed signs of deviation from the PH assumption were performed. Predicted survival plots based on the Cox PH regression model are also presented. Individuals with missing values on HADS-D (12.6 %) or other covariates were excluded from the analyses (complete case analysis, n= 34 464).

Time-varying exposure factors

Except for sex, all prognostic factors were treated as time-varying covariates in the Cox PH regression model, allowing a shift in exposure categories with increasing age for individuals who participated in two HUNT surveys. The covariate values at start of follow-up were defined according to values obtained at the HUNT participation closest in time before the age of 60 years or at the first HUNT participation at an older age. In the Kaplan-Meier survival analyses, the 'at risk' category was defined according to the most recent registered value of the

For peer review only - http://bmjopen.bmj.com/site/about/guidelines.xhtml

exposure factor during follow-up. The indicator variable for AAA was 0 (not present) until date (age) of diagnosis (recoded to 1, i.e. present). The diagnosis of AAA could occur either before, after or between two HUNT participations, depending on age at diagnosis and age at participation in a HUNT survey. A total of 27 individuals (2 women) had been diagnosed with AAA at start of follow-up from the age of 60 years, whereas an additional 556 individuals (149 women) were diagnosed during follow-up.

All statistical analyses were performed using Stata (Version IC.16.1 Stata Corp, College Station, TX). A p value <0.05 was considered significant (2-sided tests).

For peer review only - http://bmjopen.bmj.com/site/about/guidelines.xhtml

RESULTS

The distribution of the demographic and clinical characteristics of the study population at start of follow-up is presented in Table 1. A total of 583 (1.6 %) individuals (432 men, 151 women) had a diagnosed AAA, and 4 832 (13.2%) individuals reported depressive symptoms (HADS-D ≥ 8). The proportion with a HADS-D score ≥ 8 was higher in individuals with than without a diagnosed AAA (Table 1). Compared with persons without AAA (considered representative for the general population), individuals with AAA were more often men (74.1 vs. 48.4 %), current smokers (57.5 vs. 35.5 %) and reported more coronary heart disease (27.4 vs. 8.9 %) and hypertension (72.5 vs. 57.5 %). Moreover, a larger proportion in the AAA group was obese (4.7 vs 3.0 %), but no significant association with BMI category was found.

All-cause mortality in individuals with AAA and in the general population

A Kaplan Meier failure plot, with estimated proportion of deaths at different ages, for individuals with and without a diagnosed AAA, and Nelson-Aalen estimates of the cumulative hazard function, are shown in Figure 1. Individuals with AAA died at a younger age compared to the general population (log rank test, p-value < 0.001; median age at death 73.0 vs. 85.2 years). In the Cox PH regression model adjusted for age only, the hazard of death in individuals with AAA was more than three times the hazard of death in the general population (Table 2, HR 3.39, 95 % CI 3.01-3.78). The difference was less pronounced in the multivariable analysis, but the hazard remained more than twice as high in the AAA group (Table 2, HR 2.66, 95 % CI 2.39-2.97).

For peer review only - http://bmjopen.bmj.com/site/about/guidelines.xhtml

Prognostic impact of depression in individuals with AAA and in the general population

At all ages, the estimated proportions of deaths were higher among individuals with than without depressive symptoms (HADS-D ≥8 vs. >8), both in individuals diagnosed with AAA and in the general population (Figure 2). Median age at death in individuals with and without depressive symptoms in the AAA group was 70.8 and 73.6 years, whereas the corresponding numbers in the general population were 83.7 and 85.3 years.

In the age-adjusted Cox PH regression analysis, the adverse effect of depressive symptoms in individuals with AAA was rather similar to the association seen in the general population (Table 2, HR for HADS-D ≥8 vs. < 8 of 1.28 in both groups). In the analyses adjusted for age, sex, smoking, coronary heart disease (CHD), diabetes, BMI, hypertension, and civil status (Table 2) the HR value for HADS-D was closer to unity and no longer statistically significant in individuals with AAA (HR 1.15, 95%CI 0.88-1.51), whereas an adverse effect remained in the general population (HR 1.23, 95%CI 1.17-1.30). The difference in these estimates (1.15 vs. 1.23) did not reach statistical significance (p, test for interaction = 0.62). The adjustment factors that contributed the most to the reduced strength of the association with depressive symptoms were CHD, BMI and smoking. There was no single factor that explained all the flattening of the risk estimate or led to very imprecise estimates.

HR values (multivariable adjusted) for combined categories of HADS-D and AAA, using a common reference group, to allow for a direct comparison of relative risk across all subgroups, are shown in Figure 3. The adverse, though weak association with depression appeared to be rather similar in individuals with and without AAA in this analysis. The prognostic impact of AAA was considerably more pronounced than the prognostic impact of depressive symptoms

For peer review only - http://bmjopen.bmj.com/site/about/guidelines.xhtml

Age-dependent effects

The Kaplan Meier failure plot, as well as the Nelson-Aalen cumulative hazard plot (Figure 2), indicated a more pronounced negative prognostic effect from depressive symptoms in AAA individuals younger than 70 years. No such age-dependent effect of depressive symptoms was seen in the general population (Figure 2). The age-dependent pattern was confirmed in the log-log-plot used to evaluate the PH assumption in the Cox regression model (Supplemental material, Figure S1). The age-dependent pattern in the AAA group remained in the analyses adjusted for potential confounders, as illustrated in the predicted survival plots based on a multivariable adjusted Cox-PH regression models stratified on HADS-D and AAA to allow for deviation from the PH assumption (Figure 4). HR values are not calculated for stratification variables in the Cox PH model, and insufficient amount of data at the youngest ages in certain subgroups, precluded an age-specific analysis. Thus, age-specific HR values for HADS-D (≥8 vs. <8) in subgroups defined by AAA (no, yes) cannot be reported.

Log-log plot also indicated deviation from the PH assumption for BMI category. Additional analyses using BMI category as a stratification variable in the Cox regression model did not notably impact the HR values for our main study variables (AAA and HADS-D).

For peer review only

Table 1. Demographic and clinical characteristics at start of follow-up, total and by AAA

Characteristics	Total population n (%)	AAA n (%)	No AAA n (%)	P-value
**Total**	36616 (100)	583 (100)	36033 (100)	
**Sex**				<0.001
Men	17883 (48.8)	432 (74.1)	17451 (48.4)	
Women	18733 (51.2)	151 (25.9)	18582 (51.6)	
**Age at measured HADS-D**				<0.001
<60	20843 (56.9)			
60-69	9550 (26.1)			
70-79	5254 (14.3)			
≥80	969 (2.4)			
**HADS-D**				<0.001
<8	31784 (86.3)	477 (81.8)	31307 (86.9)	
≥8	4832 (13.2)	106 (18.2)	4726 (13.1)	
**Smoking**				<0.001
Never	12890 (35.9)	53 (9.2)	12837 (36.4)	
Past	12738 (35.5)	192 (33.5)	12546 (35.5)	
Current	10260 (28.6)	12546 (35.5)	9931 (28.1)	
**BMI**				0.26
<25	11198 (30.8)	177 (30.7)	11021 (30.8)	
25-29	17349 (47.8)	280 (48.5)	17069 (47.7)	
30-35	6107 (16.8)	103 (17.8)	6004 (16.8)	
≥35	1679 (4.6)	17 (3.0)	1662 (4.7)	
**CHD**	3347 (9.2)	423 (27.4)	160 (8.9)	<0.001
**Hypertension**	21124 (57.7)	423 (72.5)	20701 (57.5)	<0.001
**Diabetes**	1746 (4.8)	23 (4.0)	1723 (4.8)	0.09
**Civil status**				0.35
Cohabiting	26650 (72.9)	443 (76.0)	26207 (72.8)	
Living alone	9913 (27.1)	140 (24.0)	9773 (27.2)	

AAA = Abdominal Aortic Aneurysm, HADS = Hospital Anxiety and Depression Scale, D = depression subscale, BMI = Body Mass Index, CHD = coronary heart disease
Missing data among those with valid HADS-D (n = 36616): Smoking, n = 728 (2.6%) BMI, n = 283 (0.8%), CHD, n = 23 (0.06%), systolic and diastolic blood pressure, n = 154 (0.4%), hypertension, n = 9 (0.02%), diabetes, n = 84 (0.2%), civil status, n = 53 (0.14%).
Missing data on HADS-D, n = 5259 (12.6%).

For peer review only

Table 2 Hazard ratio (HR with 95 % CI) of death for AAA (yes vs. no) and for depressive symptoms (HADS-D ≥ 8 vs < 8) in subgroups defined by AAA (yes,no)*

	No. of deahts	Age-adjusted HR (95% CI)*	Multivariable HR (95% CI)**
**AAA**			
No	9078	1.00	1.00
Yes	350	3.39 (3.01-3.78)	2.66 (2.39-2.97)
**HADS-D**			
Individuals with AAA			
<8	283	1.00	1.00
≥8	67	1.28 (0.98-1.69)	1.15 (0.88-1.51)
Individuals without AAA			
<8	7430	1.00	1.00
≥8	1648	1.28 (1.21-1.36)	1.23 (1.17-1.30)
**P-value, interaction**		0.90	0.62

Depressive symptoms were defined as a HADS-D score ≥8.
AAA = Abdominal Aortic Aneurysm, HR = Hazard ratio, CI = Confidence Interval, HADS= Hospital Anxiety and Depression Scale.
*Based on Cox PH regression model with attained age as time scale.
**Additional adjustment for sex, coronary heart disease, diabetes mellitus, body mass index-category, smoking, hypertension, civil status. HR for AAA also adjusted for HADS-D.
Complete case analyses: Number of individuals, n= 34464.

For peer review only

For peer review only - http://bmjopen.bmj.com/site/about/guidelines.xhtml

DISCUSSION

In this large, population-based prospective study, the overall risk of death was more than twice as high in individuals with AAA compared to a general population at the same age and same risk profile. There was no clear adverse effect of depressive symptoms on all-cause mortality in individuals diagnosed with AAA after adjustment for potential confounders, whereas a negative prognostic effect remained in the general population. At ages <70 years, however, there was a tendency towards a more pronounced adverse effect of depressive symptoms in individuals with AAA. This finding was uncertain due to the low number of deaths before the age of 70 years.

The finding of a substantially higher mortality in individuals with vs. without AAA is consistent with a recent study reporting markedly lower overall long-term survival in patients undergoing successful AAA repair as compared to age-and sex-adjusted mortality rates in the general population.[27] An unfavorable co-morbidity profile commonly seen in AAA patients, including other cardiovascular diseases, as well as smoking, is probably a contributing factor to the high crude mortality rates in individuals with AAA.[28] In our study, however, the relative risk estimate remained high also after adjusting for factors associated with both AAA and increased mortality, such as sex, coronary heart disease, diabetes and smoking. We are not aware of any previous studies that have compared mortality in individuals with AAA with a general population of the same age and also same level of other risk factors. Most previous studies have almost exclusively been restricted to patients that have undergone surgery for AAA or patients deemed unfit for surgery.[27, 29-31] The present study included both surgically untreated and treated AAA patients, but we did not distinguish between these subgroups in the analyses due to lack of information on this issue.

For peer review only - http://bmjopen.bmj.com/site/about/guidelines.xhtml

To our knowledge, no previous studies have examined whether depressive symptoms on mortality differ in patients with AAA compared to a general population. The results from the present study did not confirm our initial hypothesis that a negative prognostic impact of depressive symptoms may be stronger in individuals with AAA compared to a general population. On the contrary, there was no clear association between depressive symptoms and mortality in the AAA group. The lack of association could relate to the low number of individuals with both AAA and depressive symptoms. However, we did observe a slightly more pronounced adverse effect of depressive symptoms in individuals diagnosed with AAA before the age of 70 years, whereas no such age-dependent effect was seen in the general population. This pattern may relate to the fact that some risk factors for AAA, such as a high number of pack years (smoking) and unfavorable heredity patterns have been reported to be more common in young compared to older AAA patients.[32] We did adjust for smoking habits, whereas data on heritability was not available. Due to the low number of individuals in these age groups, these results must be interpreted with caution and confirmed in other studies.

The biological mechanisms behind the increased mortality rates in individuals with AAA are yet to be clarified. Inflammation seems to be an important part of both the AAA pathogenesis and growth,[33, 34] which may relate to impact from noxious stimuli such as cigarette smoke, low density lipoprotein cholesterol and hypertension.[35] With increasing age, competent repair cells are not always available, artery repair fails and the inflammatory state increases.[1] Previous studies have shown that depression is associated with low-grade chronic inflammation.[1, 15, 36] Moreover, a negative impact from inflammatory processes on mortality in patients with CVDs and concurrent depression has been suggested.[37] Our

For peer review only - http://bmjopen.bmj.com/site/about/guidelines.xhtml

finding indicating that depressive symptoms might worsen the prognosis in younger, but not in older individuals diagnosed with AAA might be somewhat contradictory. On the other hand, it is possible that individuals who develop AAA at a younger age have faster aneurysm growth that is more susceptible to a growth-enhancing effect from depression, potentially mediated by inflammation.

Strengths and limitations

A major strength of our study is that the results are based on data from a large, population-based, prospective study. The HUNT population is a validated and well-defined homogenous cohort that represents the general population in Norway. Data on risk factors were assessed at the individual level for all study participants, enabling tight adjustment for age (1- year intervals, time scale in the survival analyses), sex and other potential confounders when estimating HR values. Repeated measurements of the exposure factors made it possible to update the risk set by use of time-varying covariates. This analytic approach reduces potential misclassification bias in the risk estimates. However, not all the individuals included in our study had participated in HUNT more than once, which is a limitation. The amount of missing data for the covariates was low. All AAA cases were verified based on a certified hospital diagnosis and dates of death were obtained from national registers with compulsory recordings. It is, however, likely that there was some underdiagnosis of AAA cases. The HUNT study does not include ultrasound examinations of the abdominal aorta. The observed 1.6% crude prevalence of AAA in our study is, however, in line with the expected rate at the time.[38] Unless a potential underdiagnosis of AAA differ by value of HADS-D, the risk estimates will not be biased. Some limitations must be considered. Most data on covariates were self-reported, including symptoms of depression, cardiovascular comorbidities and

For peer review only - http://bmjopen.bmj.com/site/about/guidelines.xhtml

For peer review only

smoking, potentially leading to information bias. Symptoms of depression were assessed through self-reported questionnaires and not by a psychiatric diagnostic interview. We had no information about antidepressant use. The low number of individuals with severe depressive symptoms in our study population could be a result from recruitment bias. Individuals with severe depression might not adhere to screening programs or attend population-based studies to the same extent as non-depressed individuals. However, such recruitment bias would likely bias our results towards the null hypothesis.

CONCLUSION

In this population-based prospective study there was no significant prognostic impact of depressive symptoms on mortality in individuals with AAA, whereas an increased risk was seen in the general population. Our results revealed a hitherto unreported higher all-cause mortality in individuals with AAA compared to a general population of the same age and same risk profile. More studies with larger AAA cohorts are needed to explore the relation between depression, AAA and mortality, and a potential heterogeneity across age groups.

Acknowledgements

The Trøndelag Health Study (The HUNT Study) is a collaboration between HUNT Research Centre, (Faculty of Medicine and Health Sciences, NTNU, Norwegian University of Science and Technology), Trøndelag County Council, Central Norway Regional Health Authority, and the Norwegian Institute of Public Health.

For peer review only - http://bmjopen.bmj.com/site/about/guidelines.xhtml

Contributorship

LN contributed to study conception and design, data and statistical analysis, interpretation of study findings and drafting and critical review of the manuscript. RH contributed to study conception and design, interpretation of study findings and drafting and critical review of the manuscript.

GA contributed to study conception, and statistical methods and analysis, interpretation of study findings and critical review of the manuscript. EM contributed to study conception and design and critical review of the manuscript. MS contributed to study conception and design, interpretation of study findings and drafting and critical review of the manuscript.

Data availability statement

No data are available

The Trøndelag Health Study (HUNT) has invited persons aged 13 - 100 years to four surveys between 1984 and 2019. Comprehensive data from more than 140.000 persons having participated at least once and biological material from 78.000 persons are collected. The data are stored in HUNT databank and biological material in HUNT biobank. HUNT Research Centre has permission from the Norwegian Data Inspectorate to store and handle these data. The key identification in the data base is the personal identification number given to all Norwegians at birth or immigration, whilst de-identified data are sent to researchers upon approval of a research protocol by the Regional Ethical Committee and HUNT Research Centre. To protect participants' privacy, HUNT Research Centre aims to limit storage of data outside HUNT databank, and cannot deposit data in open repositories. HUNT databank has precise information on all data exported to different projects and are able to reproduce these on request. There are no restrictions regarding data export given approval of

For peer review only

applications to HUNT Research Centre. For more information see: https://eur01.safelinks.protection.outlook.com/?url=http%3A%2F%2Fwww.ntnu.edu%2Fhunt%2Fdata&data=04%7C01%7Cmalin.stenman%40ki.se%7C1bc54c35ed5f4ce79e0508d8a7616b52%7Cbff7eef1cf4b4f32be3da1dda043c05d%7C0%7C0%7C637443384157146991%7CUnknown%7CTWFpbGZsb3d8eyJWIjoiMC4wLjAwMDAiLCJQIjoiV2luMzIiLCJBTiI6Ik1haWwiLCJXVCI6Mn0%3D%7C1000&sdata=GwGqQpTG4u%2FoslrgO43q64XHIpvyeEMTSCo%2ByrTn7i4%3D&reserved=0

For peer review only

Competing interests

None declared

Funding

This research has been supported by the Swedish Heart-Lung Foundation (Hultgren) grant number: 20190553 and 20180506.

Patient consent for publication

Not required

Ethics approval

The study was approved by the HUNT Study board of directors and the Regional Ethics Committee (2014/175/REK12 Midt-Norge).

Disclosures

The authors of this manuscript have no potential conflicts of interest.

Licence statement

The Submitting Author accepts and understands that any supply made under these terms is made by BMJ to the Submitting Author unless you are acting as an employee on behalf of your employer or a postgraduate student of an affiliated institution which is paying any applicable article publishing charge ("APC") for Open Access articles. Where the Submitting Author wishes to make the Work available on an Open Access basis (and intends to pay the relevant APC), the terms of reuse of such Open Access shall be governed by a Creative Commons licence.

For peer review only

For peer review only - http://bmjopen.bmj.com/site/about/guidelines.xhtml

REFERENCES

1. Sakalihasan N, Michel JB, Katsargyris A, et al. Abdominal aortic aneurysms. *Nat Rev Dis Primers* 2018;4:34.
2. Hare DL, Toukhsati SR, Johansson P, et al. Depression and cardiovascular disease: a clinical review. *Eur Heart J* 2014;35:1365-72.
3. Bath MF, Sidloff D, Saratzis A, et al. Impact of abdominal aortic aneurysm screening on quality of life. *Br J Surg* 2018;105:203-08.
4. Wanhainen A, Hultgren R, Linne A, et al. Outcome of the Swedish Nationwide Abdominal Aortic Aneurysm Screening Program. *Circulation* 2016;134:1141-48.
5. Norman PE, Jamrozik K, Lawrence-Brown MM, et al. Population based randomised controlled trial on impact of screening on mortality from abdominal aortic aneurysm. *Bmj* 2004;329:1259.
6. Ashton HA, Buxton MJ, Day NE, et al. The Multicentre Aneurysm Screening Study (MASS) into the effect of abdominal aortic aneurysm screening on mortality in men: a randomised controlled trial. *Lancet* 2002;360:1531-9.
7. Lindholt JS, Juul S, Fasting H, et al. Screening for abdominal aortic aneurysms: single centre randomised controlled trial. *Bmj* 2005;330:750.
8. Ali MU, Fitzpatrick-Lewis D, Miller J, et al. Screening for abdominal aortic aneurysm in asymptomatic adults. *J Vasc Surg* 2016;64:1855-68.
9. Timmers TK, van Herwaarden JA, de Borst GJ, et al. Long-term survival and quality of life after open abdominal aortic aneurysm repair. *World J Surg* 2013;37:2957-64.
10. de Bruin JL, Baas AF, Heymans MW, et al. Statin therapy is associated with improved survival after endovascular and open aneurysm repair. *J Vasc Surg* 2014;59:39-44.e1.
11. Bath MF, Gokani VJ, Sidloff DA, et al. Systematic review of cardiovascular disease and cardiovascular death in patients with a small abdominal aortic aneurysm. *Br J Surg* 2015;102:866-72.
12. Mykletun A, Bjerkeset O, Overland S, et al. Levels of anxiety and depression as predictors of mortality: the HUNT study. *Br J Psychiatry* 2009;195:118-25.
13. Harshfield EL, Pennells L, Schwartz JE, et al. Association Between Depressive Symptoms and Incident Cardiovascular Diseases. *Jama* 2020;324:2396-405.
14. Stenman M, Holzmann MJ, Sartipy U. Relation of major depression to survival after coronary artery bypass grafting. *Am J Cardiol* 2014;114:698-703.
15. Nemeroff CB, Goldschmidt-Clermont PJ. Heartache and heartbreak--the link between depression and cardiovascular disease. *Nat Rev Cardiol* 2012;9:526-39.
16. Daskalopoulou M, George J, Walters K, et al. Depression as a Risk Factor for the Initial Presentation of Twelve Cardiac, Cerebrovascular, and Peripheral Arterial Diseases: Data Linkage Study of 1.9 Million Women and Men. *PLoS One* 2016;11:e0153838.
17. Nyronning LA, Stenman M, Hultgren R, et al. Symptoms of Depression and Risk of Abdominal Aortic Aneurysm: A HUNT Study. *J Am Heart Assoc* 2019;8:e012535.
18. Pan A, Sun Q, Okereke OI, et al. Depression and risk of stroke morbidity and mortality: a meta-analysis and systematic review. *Jama* 2011;306:1241-9.
19. Sakalihasan N, Van Damme H, Gomez P, et al. Positron emission tomography (PET) evaluation of abdominal aortic aneurysm (AAA). *Eur J Vasc Endovasc Surg* 2002;23:431-6.

For peer review only

20. Dale MA, Ruhlman MK, Baxter BT. Inflammatory cell phenotypes in AAAs: their role and potential as targets for therapy. *Arterioscler Thromb Vasc Biol* 2015;35:1746-55.
21. Almeida OP, Ford AH, Hankey GJ, et al. Depression, antidepressants and the risk of cardiovascular events and death in older men. *Maturitas* 2019;128:4-9.
22. Cuijpers P, Vogelzangs N, Twisk J, et al. Comprehensive meta-analysis of excess mortality in depression in the general community versus patients with specific illnesses. *Am J Psychiatry* 2014;171:453-62.
23. Letnes JM, Torske MO, Hilt B, et al. Symptoms of depression and all-cause mortality in farmers, a cohort study: the HUNT study, Norway. *BMJ Open* 2016;6:e010783.
24. Krokstad S, Langhammer A, Hveem K, et al. Cohort Profile: the HUNT Study, Norway. *Int J Epidemiol* 2013;42:968-77.
25. Bjelland I, Dahl AA, Haug TT, et al. The validity of the Hospital Anxiety and Depression Scale. An updated literature review. *J Psychosom Res* 2002;52:69-77.
26. Myrtveit SM, Ariansen AM, Wilhelmsen I, et al. A population based validation study of self-reported pensions and benefits: the Nord-Trondelag health study (HUNT). *BMC Res Notes* 2013;6:27.
27. Bulder RMA, Talvitie M, Bastiaannet E, et al. Long-term Prognosis After Elective AAA Repair is Poor in Women and Men: The Challenges Remain. *Ann Surg* 2020.
28. Toghill BJ, Saratzis A, Bown MJ. Abdominal aortic aneurysm-an independent disease to atherosclerosis? *Cardiovasc Pathol* 2017;27:71-75.
29. Whittaker JD, Meecham L, Summerour V, et al. Outcome after Turndown for Elective Abdominal Aortic Aneurysm Surgery. *Eur J Vasc Endovasc Surg* 2017;54:579-86.
30. Rueda-Ochoa OL, van Bakel P, Hoeks SE, et al. Survival After Uncomplicated EVAR in Octogenarians is Similar to the General Population of Octogenarians Without an Abdominal Aortic Aneurysm. *Eur J Vasc Endovasc Surg* 2020;59:740-47.
31. Norman PE, Semmens JB, Lawrence-Brown MM, et al. Long term relative survival after surgery for abdominal aortic aneurysm in western Australia: population based study. *Bmj* 1998;317:852-6.
32. Howard DP, Banerjee A, Fairhead JF, et al. Age-specific incidence, risk factors and outcome of acute abdominal aortic aneurysms in a defined population. *Br J Surg* 2015;102:907-15.
33. Kuivaniemi H, Ryer EJ, Elmore JR, et al. Understanding the pathogenesis of abdominal aortic aneurysms. *Expert Rev Cardiovasc Ther* 2015;13:975-87.
34. Eagleton MJ. Inflammation in abdominal aortic aneurysms: cellular infiltrate and cytokine profiles. *Vascular* 2012;20:278-83.
35. Rocha VZ, Libby P. Obesity, inflammation, and atherosclerosis. *Nat Rev Cardiol* 2009;6:399-409.
36. Raedler TJ. Inflammatory mechanisms in major depressive disorder. *Curr Opin Psychiatry* 2011;24:519-25.
37. Celano CM, Huffman JC. Depression and cardiac disease: a review. *Cardiol Rev* 2011;19:130-42.
38. Ulug P, Powell JT, Sweeting MJ, et al. Meta-analysis of the current prevalence of screen-detected abdominal aortic aneurysm in women. *Br J Surg* 2016;103:1097-104.

For peer review only

Figure legends

Figure 1.
Kaplan-Meier failure plot and Nelson-Aalen cumulative hazard estimates in subgroups defined by AAA (no, yes)

Figure 2.
Kaplan-Meier failure plot and Nelson-Aalen cumulative hazard estimates in subgroups defined by HADS-D (< 8 and ≥8) and AAA (no, yes).

Figure 3.
HR values with 95 % CI for the combined categories of AAA (no, yes) and HADS-D (<8, ≥8), using a common reference group (no AAA, HADS-D < 8), plotted on logarithmic scale (base 2). The HR values are calculated in a multivariable adjusted Cox PH regression model. AAA = Abdominal Aortic Aneurysm, HADS = Hospital Anxiety and Depression Scale.

Figure 4.
Predicted survival plots based on a Cox PH regression model stratified for HADS-D and AAA (combined categories) to allow for an age-dependent effect. Curves are calculated for reference categories of adjustment factors (sex, BMI category, smoking, CHD, diabetes mellitus, hypertension and civil status). Complete case analyses.

For peer review only

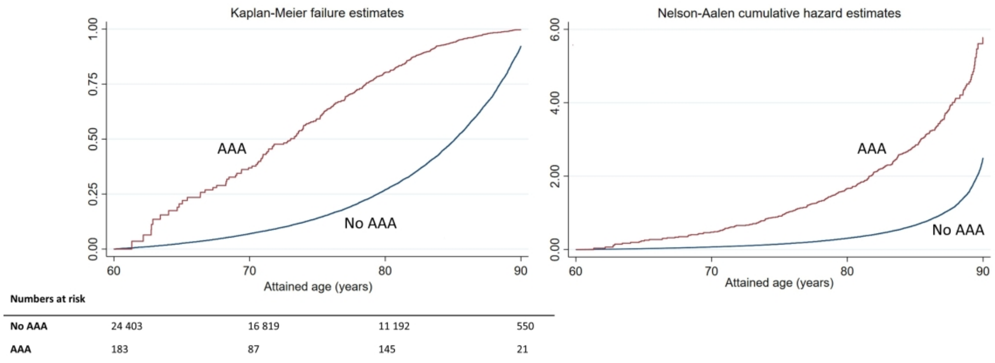

Kaplan-Meier failure plot and corresponding Nelson-Aalen cumulative hazard estimates in subgroups defined by AAA (no, yes)

338x190mm (300 x 300 DPI)

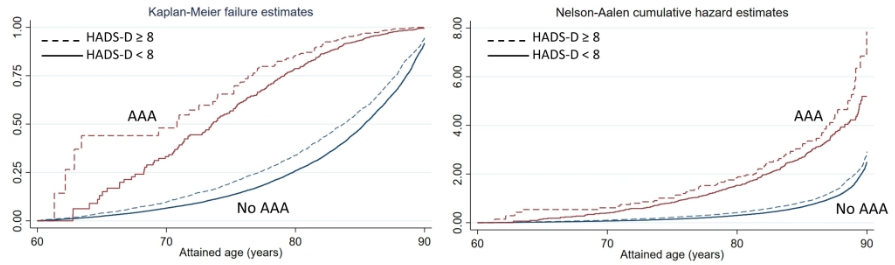

Numbers at risk

**No AAA, HADS-D<8**	19 931	13 690	8 129	320
**No AAA, HADS-D≥8**	2 559	1 986	1 462	71
**AAA, HADS-D<8**	138	68	107	10
**AAA, HADS-D≥8**	45	10	23	5

Kaplan-Meier failure plot and corresponding Nelson-Aalen cumulative hazard estimates in subgroups defined by HADS-D (< 8 and ≥8) and AAA (no, yes).

338x190mm (300 x 300 DPI)

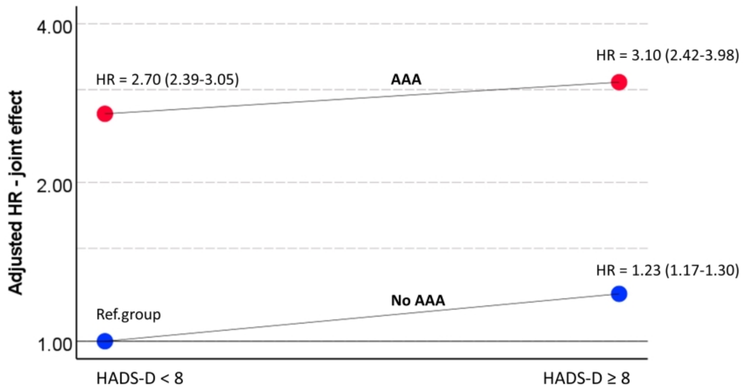

HR values with 95 % CI for the combined categories of AAA (no, yes) and HADS-D, using a common reference group (no AAA, HADS-D < 8). The results are based on the adjusted Cox PH regression model.
AAA = Abdominal Aortic Aneurysm, HADS = Hospital Anxiety and Depression Scale.

338x190mm (300 x 300 DPI)

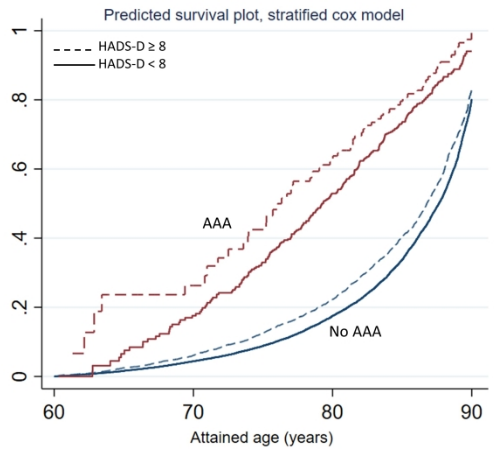

Predicted survival plots based on a Cox PH regression model stratified for HADS-D and AAA (combined categories) to allow for an age-dependent effect. Curves are calculated for reference categories of adjustment factors (sex, BMI category, smoking, CHD, diabetes mellitus, hypertension and civil status). Complete case analyses.

338x190mm (300 x 300 DPI)

Supplemental material

The prognostic impact of depressive symptoms on all-cause mortality in individuals with abdominal aortic aneurysm and in the general population. A population-based prospective HUNT Study in Norway

Linn Å. Nyrønning[1,2] Rebecka Hultgren[3,4] Grethe Albrektsen[5,6], Erney Mattsson[1,2] Malin Stenman[3,7]

[1]Department of Surgery, Vascular Surgery, St Olavs Hospital, Trondheim, Norway,

[2]Department of Circulation and Medical Imaging, NTNU, Trondheim, Norway,

[3]Department of Molecular Medicine and Surgery, Karolinska Institutet, Stockholm, Sweden,

[4]Department of Vascular Surgery, Karolinska University Hospital, Stockholm, Sweden,

[5]Department of Public Health and Nursing, NTNU, Trondheim, Norway,

[6]Department of clinical and molecular medicine, NTNU, Trondheim, Norway,

[7]Perioperative Medicine and Intensive Care Function, Karolinska University Hospital, Stockholm, Sweden

Corresponding author:

Malin Stenman

Perioperative Medicine and Intensive Care Function

Karolinska University Hospital / Karolinska Institutet

SE-171 76 Stockholm, Sweden

E-mail: Malin.Stenman@ki.se

For peer review only

Figure S1. “Log - log plot” to test the proportional hazards assumption. If the plotted lines are reasonably parallel, the proportional hazards assumption has not been violated.

For peer review only

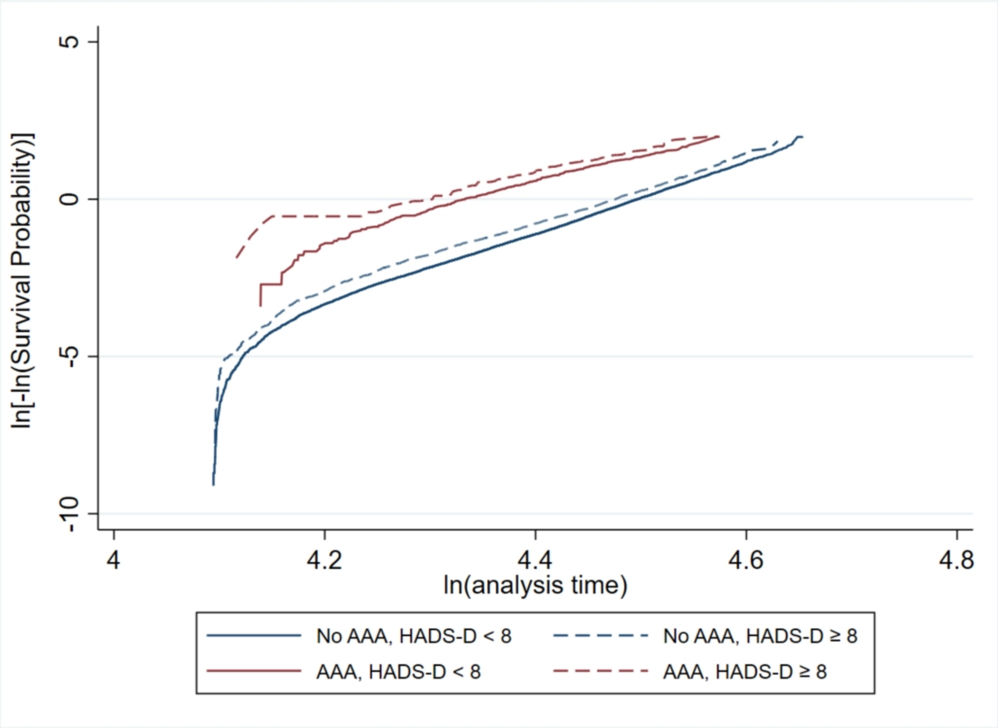

Figure S1. “Log - log plot” to test the proportional hazards assumption. If the plotted lines are reasonably parallel, the proportional hazards assumption has not been violated.

415x302mm (300 x 300 DPI)

STROBE 2007 (v4) Statement—Checklist of items that should be included in reports of *cohort studies*

Section/Topic	Item #	Recommendation	Reported on page #
**Title and abstract**	1	(*a*) Indicate the study's design with a commonly used term in the title or the abstract	1
		(*b*) Provide in the abstract an informative and balanced summary of what was done and what was found	2
**Introduction**			
Background/rationale	2	Explain the scientific background and rationale for the investigation being reported	4-5
Objectives	3	State specific objectives, including any prespecified hypotheses	5
**Methods**			
Study design	4	Present key elements of study design early in the paper	5
Setting	5	Describe the setting, locations, and relevant dates, including periods of recruitment, exposure, follow-up, and data collection	6-8
Participants	6	(*a*) Give the eligibility criteria, and the sources and methods of selection of participants. Describe methods of follow-up	6
		(*b*) For matched studies, give matching criteria and number of exposed and unexposed	6
Variables	7	Clearly define all outcomes, exposures, predictors, potential confounders, and effect modifiers. Give diagnostic criteria, if applicable	6-9
Data sources/ measurement	8*	For each variable of interest, give sources of data and details of methods of assessment (measurement). Describe comparability of assessment methods if there is more than one group	7-8
Bias	9	Describe any efforts to address potential sources of bias	9
Study size	10	Explain how the study size was arrived at	6
Quantitative variables	11	Explain how quantitative variables were handled in the analyses. If applicable, describe which groupings were chosen and why	7-9
Statistical methods	12	(*a*) Describe all statistical methods, including those used to control for confounding	8-9
		(*b*) Describe any methods used to examine subgroups and interactions	9
		(*c*) Explain how missing data were addressed	9
		(*d*) If applicable, explain how loss to follow-up was addressed	
		(*e*) Describe any sensitivity analyses	9
**Results**			

For peer review only

Participants	13*	(a) Report numbers of individuals at each stage of study—eg numbers potentially eligible, examined for eligibility, confirmed eligible, included in the study, completing follow-up, and analysed	6,7,11
		(b) Give reasons for non-participation at each stage	
		(c) Consider use of a flow diagram	
Descriptive data	14*	(a) Give characteristics of study participants (eg demographic, clinical, social) and information on exposures and potential confounders	11
		(b) Indicate number of participants with missing data for each variable of interest	14
		(c) Summarise follow-up time (eg, average and total amount)	7
Outcome data	15*	Report numbers of outcome events or summary measures over time	11-12
Main results	16	(*a*) Give unadjusted estimates and, if applicable, confounder-adjusted estimates and their precision (eg, 95% confidence interval). Make clear which confounders were adjusted for and why they were included	11-13
		(*b*) Report category boundaries when continuous variables were categorized	8, 14
		(*c*) If relevant, consider translating estimates of relative risk into absolute risk for a meaningful time period	
Other analyses	17	Report other analyses done—eg analyses of subgroups and interactions, and sensitivity analyses	11-13
**Discussion**			
Key results	18	Summarise key results with reference to study objectives	16
**Limitations**			16-19
Interpretation	20	Give a cautious overall interpretation of results considering objectives, limitations, multiplicity of analyses, results from similar studies, and other relevant evidence	19
Generalisability	21	Discuss the generalisability (external validity) of the study results	19
**Other information**			
Funding	22	Give the source of funding and the role of the funders for the present study and, if applicable, for the original study on which the present article is based	21

*Give information separately for cases and controls in case-control studies and, if applicable, for exposed and unexposed groups in cohort and cross-sectional studies.

Note: An Explanation and Elaboration article discusses each checklist item and gives methodological background and published examples of transparent reporting. The STROBE checklist is best used in conjunction with this article (freely available on the Web sites of PLoS Medicine at http://www.plosmedicine.org/, Annals of Internal Medicine at http://www.annals.org/, and Epidemiology at http://www.epidem.com/). Information on the STROBE Initiative is available at www.strobe-statement.org.

For peer review only
